# Patient-Specific Mathematical Model of the Clear Cell Renal Cell Carcinoma Microenvironment

**DOI:** 10.3390/jpm12101681

**Published:** 2022-10-09

**Authors:** Dilruba Sofia, Navid Mohammad Mirzaei, Leili Shahriyari

**Affiliations:** Department of Mathematics and Statistics, University of Massachusetts Amherst, Amherst, MA 01003, USA

**Keywords:** clear cell renal cell carcinoma (ccRCC), immune infiltration, ordinary differential equations, data-driven mathematical model, sensitivity analysis, cytokines, T-cells, macrophages, IL-6

## Abstract

The interactions between cells and molecules in the tumor microenvironment can give insight into the initiation and progression of tumors and their optimal treatment options. In this paper, we developed an ordinary differential equation (ODE) mathematical model of the interaction network of key players in the clear cell renal cell carcinoma (ccRCC) microenvironment. We then performed a global gradient-based sensitivity analysis to investigate the effects of the most sensitive parameters of the model on the number of cancer cells. The results indicate that parameters related to IL-6 have high a impact on cancer cell growth, such that decreasing the level of IL-6 can remarkably slow the tumor’s growth.

## 1. Introduction

Clear cell renal cell carcinoma (ccRCC) is a type of kidney cancer that makes up 80% of kidney cancer cases [1]. Surgery is the most common treatment for ccRCC, although it is not effective when the tumor metastasizes to other parts of the body [1,2,3]. Other treatment options, such as immunotherapy and targeted therapy, are used to regulate the growth of the tumor, and radiation therapy, thermal ablation, and cryosurgery are used to kill cancer cells [1]. However, not all patients respond well to these treatments, leading to more complications [1,2].

Mathematical models can provide valuable information about the complex interactions in biological systems, such as cancer [4,5,6,7,8,9,10,11,12,13]. For example, Pillis et al. created a mathematical model on the effects of regulatory T-cells (Tregs) on ccRCC treatment via a drug called sunitinib, which resulted in an improvement in controlling cancer in 40% of patients compared to patients without treatment [14]. Since vascular endothelial growth factor (VEGF) helps in angiogenesis in tumors, another study by Sharma et al. studied the effectiveness of the VEGF receptor 2 (VEGFR2) inhibitor in renal cell carcinoma (RCC) patients and found that an inhibitor compound named SCHEMBL469307 is the most effective [15]. In addition, complex data-driven mathematical models of other cancers, such as breast cancer, osteosarcoma, and colon cancer, were developed by Mohammad Mirzaei et al., Kirshtein et al., and Le et al. to investigate the interactions between the important immune cells and cytokines involving many key cells and molecules corresponding to the particular type of cancer and found that the interactions between the cells and molecules are important to understand the dynamics of cancer growth [16,17,18].

The tumor microenvironment, including its cytotoxicity, plays an essential role in inhibiting cancer proliferation [19]. Many classical studies focused on combination treatments, including cytokines, such as interferon-γ (IFN-γ), and IL-2 [20,21,22]. However, these approaches are becoming rather obsolete [23]. More recently, treatments using anti-PD-1/PD-L1 have shown great promise in treating patients [24] by increasing the cytotoxicity of the tumor microenvironment. The programmed cell death proteins (PD-1 or PD-2) can bind with programmed cell death ligands (PD-L1 or PD-L2) and inhibit the killing effects of cytotoxic and natural killer cells [25,26,27]. PD-1 and PD-2 proteins are expressed by cytotoxic cells, and PD-L1 and PD-L2 are expressed in cancer cells in the ccRCC microenvironment as adaptation mechanisms to cope with cytotoxicity [25,26,27]. On the other hand, CD8+ T-cells are promoted by helper T-cells, dendritic cells, interleukin-2 (IL-2), and interleukin-12 (IL-12), which could negate the blockade created by the bonding of PD-1/2 with PDL-1/2 [27,28]. IFN-γ is another molecule secreted by CD8+ T-cells, helper T-cells, and dendritic cells, which can inhibit cancer cell proliferation [29]. When tumor cells die, many of them go through cell necrosis and release damage-associated molecular pattern molecules (DAMPs), such as high mobility group box-1 (HMGB1) [19,30,31,32]. HMGB1 is known as a nuclear weapon in the tumor microenvironment as it can do many things, such as induce inflammation, interact with dendritic cells to promote anti-tumor T-cells, and so on [19,30,32,33,34]. Although metastasis can happen throughout the human body, metastatic tumors resemble their corresponding primary tumors [35].

The ccRCC microenvironment has the highest levels of immune cell infiltration among all epithelial cancer types, making it a pro-inflammatory environment [36]. Infiltrating T-cells, such as cytotoxic and helper T-cells, play essential roles in controlling the tumor by targeting antigenic tumor cells [37]; thus, treatments (such as sunitinib and pazopanib), which directly target these T-cells in ccRCC, have been dominant for a long time. However, it has been observed that tumor microenvironment heterogeneity can significantly affect the outcomes of these treatments. A study on patients in the phase III trial receiving sunitinib or pazopanib shows poor outcomes for a cluster of patients with a high immune infiltration (especially macrophages) and significantly higher PD-L1 expression on tumor cells compared to the other clusters [38]. Another study based on VEGFR TKI therapy on 53 metastatic ccRCC patients revealed undesirable outcomes in a group of patients whose tumors had an intense Th1-oriented inflammatory and suppressive immune environment, with high levels of PD-1/PD-L1 [39]. These observations emphasize the sensitivity of clinical treatment responses to tumors’ immune profiles.

**Figure 1:**
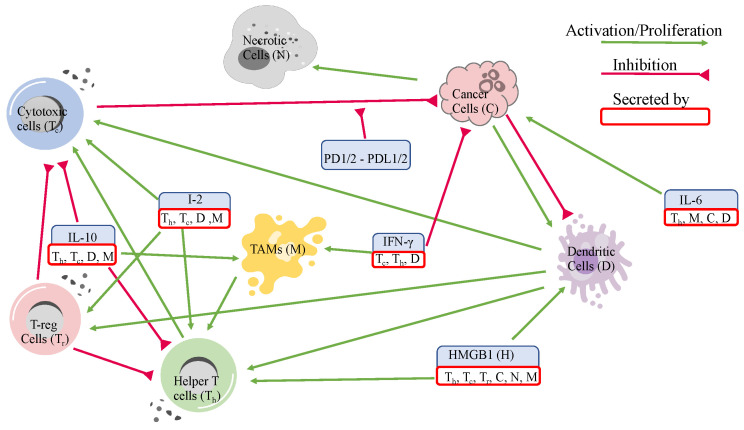
Tumor microenvironment interaction network of ccRCC.

This paper investigates the dynamics of tumors based on their immune patterns by developing an ODE model that considers the interaction network of key players that we mentioned above (see Figure 1). We clustered the tumors based on their immune patterns and compares their dynamics. More importantly, we found the most sensitive parameters for each cluster of tumors. The parameters in this model were estimated using the steady state assumptions and data acquired from gene expression data from The Cancer Genome Atlas (TCGA), separately for each cluster of tumors. The result was a data-driven patient-specific ODE model aimed at understanding the impacts of immune patterns and their interaction network in tumor progression. Moreover, regarding its capability to extend to a treatment model, one can treat the immune cells as boundary variables and integrate this model as a compartment for larger-scale models. Furthermore, by including metabolites, such as nutrients and oxygen, one can extend the model to incorporate angiogenesis. However, at this stage, we avoid introducing more complexity to this model.

## 2. Materials and Methods

### 2.1. Variables and Network

As mentioned in the introduction, ccRCC has the highest infiltration of cytotoxic cells, dendritic cells, Th1, and macrophages among all of the other epithelial cancers, and low levels of regulatory T-cells and Th2 [36]; the response to treatments are associated with the percentage of these cells in the tumors. Therefore, including Th1 and Th2 together as helper T-cells, we used these key cells in addition to cancer and necrotic cells, and the most important cytokines secreted by them as our model variables.

Helper T-cells, interleukin-12 (IL-12), and interleukin-2 (IL-2) help the proliferation of cytotoxic cells, but regulatory T-cells (T-reg cells) inhibit both helper T-cells and cytotoxic cells [28,32,40,41,42,43]. Furthermore, the cytokine interleukin-10 (IL-10) inhibits both helper T-cells and cytotoxic cells, limiting their ability to grow while promoting macrophages [44,45,46]. Cytotoxic cells express programmed cell death proteins (PD-1 or PD-2) and cancer cells as adaptation mechanisms express programmed cell death ligands (PD-L1 or PD-l2) [25,26,27,47]. When PD-1 or PD-2 on cytotoxic cells bind to the ligands PD-L1 or PD-L2, this initiates apoptosis in cytotoxic cells, reducing their ability to kill cancer cells [25,26,27,47]. Cancer cells also adapt to the environment by secreting interleukin-6 (IL-6), which also promotes cancer cells [48,49,50].

In addition, both cancer and necrotic cells release relatively large portions of HMGB1 that helps promote dendritic cells and helper T-cells [30,31,33,51]. Dendritic cells then promote both helper T-cells and cytotoxic cells [19,30,31,51,52]. To demonstrate the interactions among the immune cells, cytokines, cancer, and necrotic cells, we developed an interaction network in Figure 1 and provide the list of variables and how we derived them in Table 1.

### 2.2. Mathematical Model

We derived an ordinary differential equation (ODE) for each variable in the model using λ to denote the growth/promotion rates and δ to denote the decay/inhibition rates of the associated cells and molecules. In the case of promotion and inhibition, the second subscript refers to the promoter or inhibitor, and the first subscript is the cell or molecule subjected to promotion or inhibition.

#### 2.2.1. Cells

**Helper T-cells ($T_{h}$)**: Helper T-cells are activated by macrophages, dendritic cells, HMGB1, and IL-12 [34,53,54,55]. All of the T-cells in the model differentiate from naive T-cells. However, this differentiation happens mostly outside of the microenvironment. For simplicity, we added naive T-cells to the model to prevent blow-ups in the population of active T-cells without having to introduce nonlinearity in their corresponding ODEs [16,56]. Regulatory T-cells inhibit helper T-cells, and IL-10 [45,46]. Therefore, the dynamics of helper T-cells are modeled by the following equation.

(1) $$\begin{array}{cc} \frac{d[T_{h}]}{dt} & =\left(\lambda_{T_{h}M}\left[M\right]+\lambda_{T_{h}D}\left[D\right]+\lambda_{T_{h}H}\left[H\right]+\lambda_{T_{h}I_{2}}\left[I_{2}\right]\right)\left[T_{N}\right]\\ & -\left(\delta_{T_{h}T_{r}}\left[T_{r}\right]+\delta_{T_{h}IL_{10}}\left[IL_{10}\right]+\delta_{T_{h}}\right)\left[T_{h}\right]. \end{array}$$

**Cytotoxic cells ($T_{c}$)**: Cytokines, such as IL-2 and IL-12 activate CD8+ T-cells and promote NK cells [57,58]. Dendritic cells also promote the cytotoxic cells [53,59], and so do helper T-cells [60]. On the other hand, interferon-γ can either kill cancer cells or promote CD8+ T-cells [25,29,58,61]. T-reg cells control the population of CD8+ T-cells [19,41,62], and IL-10 reduces the cytotoxicity of CD8+ T-cells [44,45,46]. Hence, we have the following equation for the dynamics of cytotoxic T-cells.

(2) $$\begin{array}{cc} \frac{d[T_{c}]}{dt} & =\left(\lambda_{T_{c}T_{h}}\left[T_{h}\right]+\lambda_{T_{c}I_{2}}\left[I_{2}\right]+\lambda_{T_{c}D}\left[D\right]+\lambda_{T_{c}I_{\gamma }}\left[I_{\gamma }\right]\right)\left[T_{N}\right]\\ & -\left(\delta_{T_{c}IL_{10}}\left[IL_{10}\right]+\delta_{T_{c}T_{r}}\left[T_{r}\right]+\delta_{T_{c}}\right)\left[T_{c}\right]. \end{array}$$

**Regulatory T-cells ($T_{r}$):** IL-12 and IL-2 activate regulatory T-cells [28,41,43,55,63]. We only assume natural death for the regulatory T-cells since the model has no major inhibitors for this cell type. Thus, we model the dynamics of T-reg cells by

(3) $$\begin{array}{c} \frac{d[T_{r}]}{dt}=\left(\lambda_{T_{r}D}\left[D\right]+\lambda_{T_{r}I_{2}}\left[I_{2}\right]\right)\left[T_{N}\right]-\delta_{T_{r}}\left[T_{r}\right]. \end{array}$$

**Naive T-cells ($T_{N}$):** As mentioned earlier, naive T-cells are not a part of the tumor microenvironment. They differentiate into other T-cell types mostly before the microenvironment immune infiltration [34,56]. We modeled naive T-cells starting with a constant growth rate and then deducted the differentiation rate of other T-cells.

(4) $$\begin{array}{cc} \frac{d[T_{N}]}{dt} & =A_{T_{N}}-\left(\lambda_{T_{h}M}\left[M\right]+\lambda_{T_{h}D}\left[D\right]+\lambda_{T_{h}H}\left[H\right]+\lambda_{T_{h}I_{2}}\left[I_{2}\right]\right)\left[T_{N}\right]\\ & -\left(\lambda_{T_{c}T_{h}}\left[T_{h}\right]+\lambda_{T_{c}I_{2}}\left[I_{2}\right]+\lambda_{T_{c}D}\left[D\right]+\lambda_{T_{c}I_{\gamma }}\left[I_{\gamma }\right]\right)\left[T_{N}\right]\\ & -\left(\lambda_{T_{r}D}\left[D\right]+\lambda_{T_{r}I_{2}}\left[I_{2}\right]+\delta_{T_{N}}\right)\left[T_{N}\right]. \end{array}$$

**Macrophages (*M*):** Tumor-associated macrophages have M1 and M2 phenotypes [44,64,65]. However, since the phenotypes can change between one another, for simplicity, we combined them into one variable *M* [44]. Macrophages are activated by interferon-γ and IL-10 [64,65,66,67] and helper T-cells [61].

(5) $$\begin{array}{cc} \frac{d[M]}{dt} & =\left(\lambda_{MT_{h}}\left[T_{h}\right]+\lambda_{MI_{\gamma }}\left[I_{\gamma }\right]+\lambda_{MIL_{10}}\left[IL_{10}\right]\right)\left[M_{N}\right]-\delta_{M}\left[M\right]. \end{array}$$

**Naive macrophages ($M_{N}$):** Since macrophages are derived from naive macrophages [44], we used an approach similar to the naive T-cells to model naive macrophages.

(6) $$\begin{array}{cc} \frac{d[M_{N}]}{dt} & =A_{M_{N}}-\left(\lambda_{MT_{h}}\left[T_{h}\right]+\lambda_{MI_{\gamma }}\left[I_{\gamma }\right]+\lambda_{MIL_{10}}\left[IL_{10}\right]+\delta_{M_{N}}\right)\left[M_{N}\right]. \end{array}$$

**Dendritic cells (*D*):** Dendritic cells are activated by HMGB1 [19,68,69]. Moreover, dendritic cells can be activated or suppressed by cancer cells [51,68].

**Naive dendritic cells ($D_{N}$):** Mature dendritic cells are derived from naive dendritic cells [68,69,70]. So, we modeled naive dendritic cells similar to naive T-cells and naive macrophages.

(7) $$\begin{array}{c} \frac{d[D]}{dt}=\left(\lambda_{DH}\left[H\right]+\lambda_{DC}\left[C\right]\right)\left[D_{N}\right]-\left(\delta_{DC}\left[C\right]+\delta_{D}\right)\left[D\right] \end{array}$$

(8) $$\begin{array}{c} \frac{d[D_{N}]}{dt}=A_{D_{N}}-\left(\lambda_{DH}\left[H\right]+\lambda_{DC}\left[C\right]+\delta_{D_{N}}\right)\left[D_{N}\right] \end{array}$$

**Cancer cells (*C*):** Although cancer cells grow rapidly, their growth can be affected by the lack of space or nutrients. Therefore, we added a logistic model term $\left[C\right]\left(1-\frac{\left[C\right]}{C_{0}}\right)$ to control the population of cancer cells corresponding to a carrying capacity $C_{0}$. Cancer cells are also activated by IL-6 [49,71] and deactivated by CD8+ T-cells, NK cells [47,50,56], and interferon-γ [29,61]. Moreover, cytotoxic cell apoptosis happens when the programmed cell death proteins (PD-1 and PD-2) expressed by CD8+ T-cells attach to the programmed cell death ligands (PD-L1 and PD-L2) expressed by cancer cells [26,27]. This way, cancer cells can avoid CD8+ T-cell cytotoxicity. We combined the concentrations of PD-1 and PD-2 and denote them by [PD]; we denote PD-L1 and PD-L2 together as [PDL]. We make their concentrations proportional to the cells they are expressed by. So, we assume that the concentrations of PD-1/PD-2 and PDL-2 are proportional to cytotoxic and cancer cells, respectively.

(9) $$\begin{array}{c} \left[PD\right]=\alpha_{T_{c}}\left[T_{c}\right],\;and\;\left[PDL\right]=\beta_{C}\left[C\right]. \end{array}$$

We model the cancer cell population by the following.

(10) $$\begin{array}{cc} \frac{d[C]}{dt} & =\left(\lambda_{C}+\lambda_{CIL_{6}}\left[IL_{6}\right]\right)\left(1-\frac{\left[C\right]}{C_{0}}\right)\left[C\right]\\ \; & -\left(\delta_{CT_{c}}\left[T_{c}\right]\cdot \left(\frac{1}{1+\alpha_{T_{c}}\left[T_{c}\right]\cdot \beta_{C}\left[C\right]}\right)+\delta_{CI_{\gamma }}\left[I_{\gamma }\right]+\delta_{C}\right)\left[C\right] \end{array}$$

**Necrotic cells (*N*):** Necrosis is a process in which the dead cells are not cleared out of the body or the system, unlike apoptosis, autophagy, etc. [30,33]. In a tumor site, necrosis happens mostly through cancer cell death; thus, we modeled the necrotic cell populations by taking a proportion of cancer cell death and a much smaller decay rate [35].

(11) $$\begin{array}{c} \frac{d[N]}{dt}=\alpha_{NC}\left(\delta_{CT_{c}}\left[T_{c}\right]\cdot \left(\frac{1}{1+\alpha_{T_{c}}\left[T_{c}\right]\cdot \beta_{C}\left[C\right]}\right)+\delta_{CI_{\gamma }}\left[I_{\gamma }\right]+\delta_{C}\right)\left[C\right]-\delta_{N}\left[N\right] \end{array}$$

#### 2.2.2. Molecules and Proteins

**IFN-γ ($I_{\gamma }$):** Interferon-γ is mainly secreted by cytotoxic and NK cells, helper T-cells, and dendritic cells [16,29,57].

(12) $$\begin{array}{c} \frac{d[I_{\gamma }]}{dt}=\lambda_{I_{\gamma }T_{c}}\left[T_{c}\right]+\lambda_{I_{\gamma }T_{h}}\left[T_{h}\right]+\lambda_{I_{\gamma }D}\left[D\right]-\delta_{I_{\gamma }}\left[I_{\gamma }\right] \end{array}$$

**HMGB1 (*H*):** The molecule HMGB1 is produced by helper T-cells, cytotoxic cells, NK cells, regulatory T-cells, necrotic cells, and macrophages [30,31,34,52].

(13) $$\begin{array}{c} \frac{d[H]}{dt}=\lambda_{HT_{c}}\left[T_{c}\right]+\lambda_{HT_{r}}\left[T_{r}\right]+\lambda_{HT_{h}}\left[T_{h}\right]+\lambda_{HN}\left[N\right]+\lambda_{HM}\left[M\right]+\lambda_{HC}\left[C\right]-\delta_{H}\left[H\right] \end{array}$$

**Interleukin-10 ($IL_{10}$):** IL-10 is produced by helper T-cells, cytotoxic cells, dendritic cells, and macrophages [44,45,46].

(14) $$\begin{array}{c} \frac{d[IL_{10}]}{dt}=\lambda_{IL_{10}T_{h}}\left[T_{h}\right]+\lambda_{IL_{10}T_{c}}\left[T_{c}\right]+\lambda_{IL_{10}D}\left[D\right]+\lambda_{IL_{10}M}\left[M\right]-\delta_{IL_{10}}\left[IL_{10}\right]. \end{array}$$

**Interleukin-2 and interleukin-12 ($I_{2}$):** IL-2 is mainly produced by CD4+ helper T-cells [58,72] and NK cells [57]. Moreover, IL-12 is secreted by helper T-cells, cytotoxic cells, dendritic cells, and macrophages [18,43,57]. As both IL-2 and IL-12 have the same functionalities, we combine them into one variable, such as $I_{2}$.

(15) $$\begin{array}{c} \frac{d[I_{2}]}{dt}=\lambda_{I_{2}T_{c}}\left[T_{c}\right]+\lambda_{I_{2}T_{h}}\left[T_{h}\right]+\lambda_{I_{2}D}\left[D\right]+\lambda_{I_{2}M}\left[M\right]-\delta_{I_{2}}\left[I_{2}\right] \end{array}$$

**Interleukin-6 ($IL_{6}$):** IL-6 is mainly secreted by cancer cells [48,49,64]. CD4+ helper T-cells, macrophages, and dendritic cells [16,18,48,49,50,65].

(16) $$\begin{array}{c} \frac{d[IL_{6}]}{dt}=\lambda_{IL_{6}C}\left[C\right]+\lambda_{IL_{6}M}\left[M\right]+\lambda_{IL_{6}T_{h}}\left[T_{h}\right]+\lambda_{IL_{6}D}\left[D\right]-\delta_{IL_{6}}\left[IL_{6}\right]\; \end{array}$$

Hence, we have 15 ODEs in the system and 67 parameter values to determine to solve the system.

### 2.3. Data Preparation

**Figure 2:**
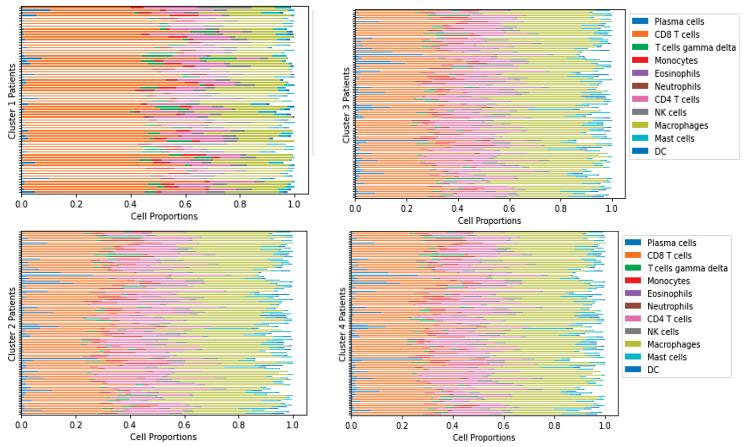
Immune cell frequencies of each cluster.

We used gene expression data of ccRCC from The Cancer Genome Atlas (TCGA) along with the immune classification by Su et al. [2] where the TCGA gene expression was normalized, and CIBERSORTx B-mode was used to derive the cell fractions [73]. The patient classification was conducted using unsupervised K-means clustering, resulting in four groups based on each cell type proportion, such as T-cells, B-cells, macrophages, dendritic cells, etc. [2]. Due to the lack of time course data, we represent each cluster of patients as one virtual patient as their immune patterns are similar. We consider the smallest tumor in each cluster to represent the first stage and the largest tumor to represent the last stage of progression over time. After clustering, the cell proportions from the CIBERSORTx result and the protein and molecule concentrations from the normalized gene expression data were derived from [2], according to the variable combination in the model described in Table 1. We only considered patient data with less than 0.5 *p*-values from the CIBERSORTx result. The cell frequencies derived from CIBERSORTx for each cluster are given in Figure 2, produced by TumorDecon software [74].

We determined the actual cell population based on the intermediate tumor size described in the TCGA clinical data of renal cell carcinoma patients [73]. We chose the ratios of immune cells to cancer cells to necrotic cells as 0.3:0.6:0.1, as was done in [18]. We assume that the epithelial cell density is $4.5\times 10^{4}$ cell/cm ${}^{3}$ in the cancer microenvironment [16,17,18,75]. We also let the average cell density scale be $\alpha =4.5\times 10^{4}$ across all ccRCC patients. We calculate the total cell number (TCN) of each patient *P* by

(17) $$\begin{array}{c} TCN=\alpha \frac{\mathrm{tumor}\;\mathrm{size}\;\mathrm{of}\;P}{\frac{1}{K}\sum_{\mathrm{all}\;P}\mathrm{tumor}\;\mathrm{size}\;\mathrm{of}\;P} \end{array}$$

where K=346 is the total number of patients having primary tumors from the original data. Then the total immune cell (TIC) population is calculated by,

(18) $$\begin{array}{c} TIC=0.3\alpha \frac{\sum_{\mathrm{all}\;\mathrm{cell}}\mathrm{Immune}\;\mathrm{cell}\;\mathrm{ratio}\;\mathrm{of}\;P}{\frac{1}{K}\sum_{\mathrm{all}\;P}\mathrm{Immune}\;\mathrm{cell}\;\mathrm{ratio}\;\mathrm{of}\;P} \end{array}$$

so

(19) $$\begin{array}{c} C=\frac{6}{7}\left(TCN-TIC\right),\;\mathrm{and}\;\;N=\frac{C}{6}. \end{array}$$

We let the cells and cytokines of the smallest tumor in each cluster be the initial conditions of the system and nondimensionalized the variables by dividing them by their steady state values. The nondimensional initial conditions are given in Table 2 and the steady state values are given in Table 3.

### 2.4. Parameter Estimation

We have 15 ODEs and 67 parameters, but there is not enough biological information to estimate all of the parameter values specific to ccRCC. However, we collected and derived some of the decay rates, such as $\delta_{T_{h}}$, $\delta_{T_{c}}$, $\delta_{T_{r}}$, $\delta_{T_{N}}$, $\delta_{M}$, $\delta_{D}$, $\delta_{I_{\gamma }}$, $\delta_{H}$, $\delta_{IL_{10}}$, $\delta_{I_{2}}$, and $\delta_{IL_{6}}$ by using the formula $\delta_{X}=\frac{\log(2)}{t_{1/2}^{X}}$ where $\delta_{X}$ is the decay rate of the corresponding variable *X* and $t_{1/2}^{X}$ is its half-life [16,17,18]. We took the average for the variables referring to combined quantities and then computed $\delta_{X}$. For instance, the half-life of IL-2 is approximately 7 min [76] and the half-life of IL-12 is almost 3.6 h [16], so we took the average of these half-lives to form the half-life of $I_{2}$ (i.e., $t_{1/2}^{I_{2}}=7.743\times 10^{-2}$ days), which gives us $\delta_{I_{2}}=2.238$$\frac{\mathrm{molecules}}{\mathrm{day}}$. The following are the decay rate values that were either collected from [16,18] or calculated based on half-lives of the variables,

$$\begin{array}{cccccccc} \delta_{T_{h}} & =0.231 & \delta_{T_{c}} & =0.406 & \delta_{T_{r}} & =0.231 & \delta_{T_{N}} & =9.49\cdot 10^{-4}\\ \delta_{M} & =1.98\cdot 10^{-2} & \delta_{D} & =0.277 & \delta_{I_{\gamma }} & =33.3 & \delta_{H} & =18\\ \delta_{IL_{10}} & =4.62 & \delta_{I_{2}} & =2.238 & \delta_{IL_{6}} & =1.07. \end{array}$$

We estimate the remaining parameters of the model by assuming the large tumors are at a steady state and utilize their data. The ODEs in Section 2.2 provide 15 algebraic equations with 67 unknowns at the steady state. Given the 11 decay rates determined above, we need to find the values for 41 unknown parameters. This system is heavily under-determined, and it is impossible to extract a unique parameter set in its current state. However, we can add some biologically feasible mathematical assumptions to remedy this issue. These assumptions simply describe a relationship between activation or inhibition of a certain cell or cytokine by other cells or molecules. This will create a system of algebraic equations at the steady state, uniquely solvable for the parameter values. See Appendix A.1 for more details on this process. We included the nondimensional parameter values in Table A3. Furthermore, given that our extra assumptions are mostly of a mathematical nature, we will assess their effects on the model dynamics by scaling them.

### 2.5. Sensitivity Analysis

Since there is not enough biological evidence to estimate all of the parameter values of the ccRCC model, the limitations of unknown parameter estimations must be considered when using the result of the dynamics. We performed a global gradient-based sensitivity analysis to assess our estimations by scaling the 34 parameter assumptions in Appendix A.1. We carried out 5000 scalings for each parameter, leaving us with 34×5000 = 170,000 variations of parameters, which is significant but still a limited number. When performing the sensitivity analysis, we used the nondimensionalized system explained in Appendix A.2 to keep the computations stable. The sensitivity level of an ODE system, such as $\frac{d\bar{X}}{dt}=F\left(\bar{X},\hat{\theta },t\right)$, where $\hat{\theta }=\langle \theta_{1},\theta_{2},\ldots ,\theta_{N}\rangle$ represents the parameter vector, is calculated by,

$$s_{i}=\frac{d\bar{X^{*}}}{d\theta_{i}}\;\mathrm{for}\;i=1,2,\cdots ,N,$$

where $\bar{X^{*}}$ is the solution of the system at the steady state. In this paper, we calculated the sensitivity of cancer and total cells to all the parameters at their steady states. For each variable $\bar{X^{*}}$, we obtained the sensitivity vector for the system by differentiating it with respect to $\theta_{i}$ and setting $F(\bar{X^{*}},\hat{\theta })=0$. We obtain the formula

$$s=\frac{d\bar{X^{*}}}{d\hat{\theta }}=-\left(\nabla F{\left(\bar{X},\hat{\theta }\right)}^{-1}\right)\left(\frac{\partial F(\bar{X^{*}},\hat{\theta })}{\partial \hat{\theta }}\right)$$

where $\nabla F{\left(\bar{X},\hat{\theta }\right)}^{-1}$ is the numerically approximated inverse Jacobian of *F* with respect to $\bar{X}$ [18]. Then, we followed the methodology for global sensitivity given in [18].

## 3. Results

### 3.1. Dynamics

**Figure 3:**
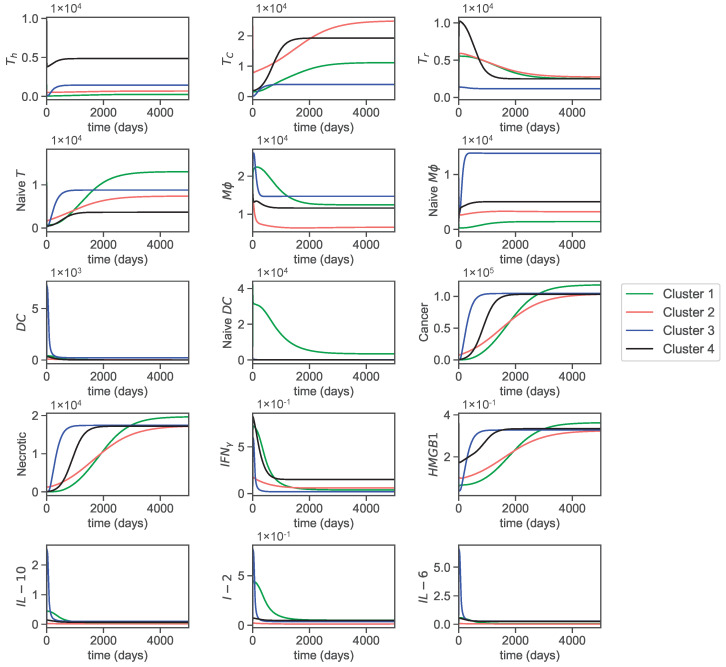
Dynamics of all variables over 5000 days.

The dynamics of the cells and molecules over 5000 days are presented in Figure 3.

The helper T-cell population decreases at the beginning and eventually increases to reach a steady state in clusters 3 and 4. In cluster 2, it increases, and in cluster 1, it decreases to reach a steady state within the first few days. As a result, the helper T-cell populations in clusters 1 and 2 remain constant almost all of the time.

Cytotoxic cells in all clusters decrease in the first few days but eventually increase in population to reach a steady state. Furthermore, cytotoxic cells in cluster 2 start with the highest population and achieve the highest saturation level. This is followed by cluster 4, with the second highest saturation level, where the cells grow faster and reach a steady state in around 1500 days. In cluster 3, cytotoxic cells reach the lowest saturation level, leaving cluster 1 with the second lowest saturation level. Overall, the steady state populations and the time points that each cluster reaches steady states are noticeably different in each cluster.

T-reg cells in clusters 1, 2, and 4 increase quickly from their initial conditions and decrease to reach a steady state. T-reg cells in cluster 4 reach a steady state at around 1500 days and clusters 1 and 2 reach a steady state at around 3000 days. In cluster 3, T-reg cells decrease and reach a steady state faster than in any other cluster. Furthermore, the slow increase in cytotoxic cells in clusters 1 and 2 could be due to regulations by T-reg cells that slowly decrease in these clusters. Similarly, the faster growth in cluster 4 of cytotoxic cells could be related to the fast decrease of T-reg cells in cluster 4.

Naive T-cells in cluster 1 attain the highest steady state population, followed by 3, 2, and 4, respectively. In clusters 1 and 2, naive T-cells increase slowly and reach a steady state of around 2000 days, whereas clusters 3 and 4 stabilize before and after 1000 days.

Macrophages (Mϕ) in clusters 1 and 3 initially increase and then soon start to decrease to reach a steady state, but in clusters 2 and 4, they decrease to reach a steady state. Naive macrophage growth in all clusters does almost the opposite of macrophages, which could be because macrophages are derived from naive macrophages. The overall trend for macrophages is that they decrease to reach a steady state in all clusters, while naive macrophages increase to reach a steady state.

The overall trend for both mature and naive dendritic cells is to decrease to reach a steady state. However, dendritic cells in cluster 3 initially increase quickly and drastically and then suddenly decrease. The same happens to naive dendritic cells in cluster 1. Moreover, cluster 1 stands out in naive dendritic cells by achieving comparatively higher steady state values than the others.

Cancer and necrotic cells exhibit exponential growth until they reach a steady state and have similar curves, as necrotic cells are produced at a rate proportional to cancer cell decay. Cancer cells in cluster 1 attain the highest steady state population. Cluster 3 stands out by growing the fastest and requiring less time to reach a steady state, which could be related to its low level of cytotoxic cells (especially since cytotoxic cells also reach a steady state at around 1000 days in this cluster). Cluster 2 has the slowest growth despite having the highest initial population. Clusters 2, 3, and 4 all achieve a similar steady state population sooner or later within 5000 days. The slow growth in cluster 2 can also be attributed to the significantly high cytotoxic levels. Overall, we can see a clear correlation between cancer progression and cytotoxicity levels.

Interferon-γ initially increases but starts to decrease to reach a steady state in all clusters. HMGB1 concentration drops at the beginning and then increases to reach a similar steady state in all clusters. Cluster 1, as with cancer and necrotic cells, achieves the highest steady state concentration in HMGB1. It is consistent with our assumptions of parameters, as HMGB1 is mainly secreted by cancer and necrotic cells in the cancer microenvironment. IL-10 concentrations in clusters 1, 3, and 4 increase initially but decrease within a few days. However, cluster 3 concentration of IL-10 increases rapidly to a higher concentration than other clusters and decreases to reach a similar steady state concentration. The cluster 1 concentration of IL-10 reaches a steady state later than other clusters (around 700 days). Cluster 2’s IL-10 concentration decreases to reach the lowest steady state concentration among all clusters. IL-2 and IL-12 concentrations in clusters 1, 3, and 4 also increase at the beginning, then decrease to reach a steady state. As with IL-10, cluster 3 concentration $I_{2}$ attains its maximum concentration very fast and decreases to reach a steady state concentration. In cluster 1, it follows a similar trend as IL-10 and slowly reaches a steady state at around 2000 days. Finally, the concentration of IL-6 in clusters 1, 3, and 4 increases and then decreases to reach a steady state. However, the concentration of IL-6 in cluster 2 remains constant for the most part.

Since we assume that the concentrations of PD−1/2 are proportional to cytotoxic cells and the concentration of PDL−2 is proportional to cancer cells, we refrain from including their dynamics.

### 3.2. Sensitivity

**Figure 4:**
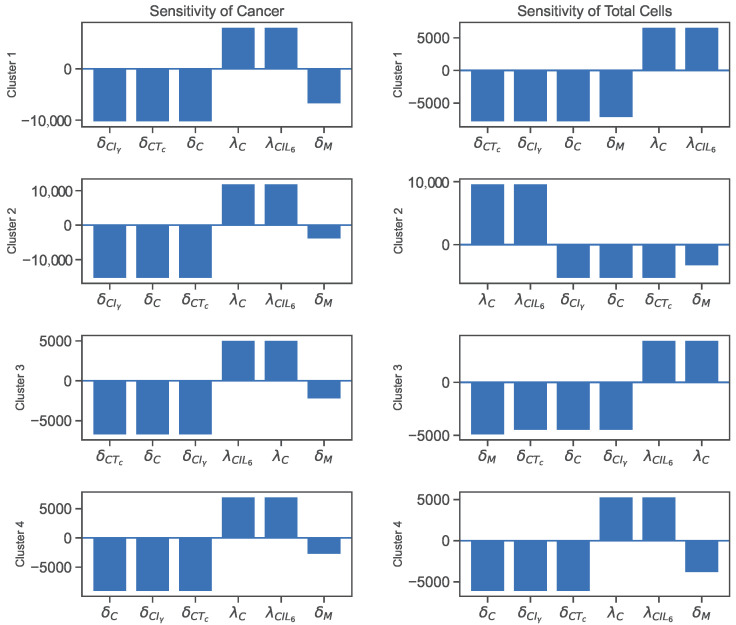
Sensitivity analysis results for cancer and total cells.

**Figure 5:**
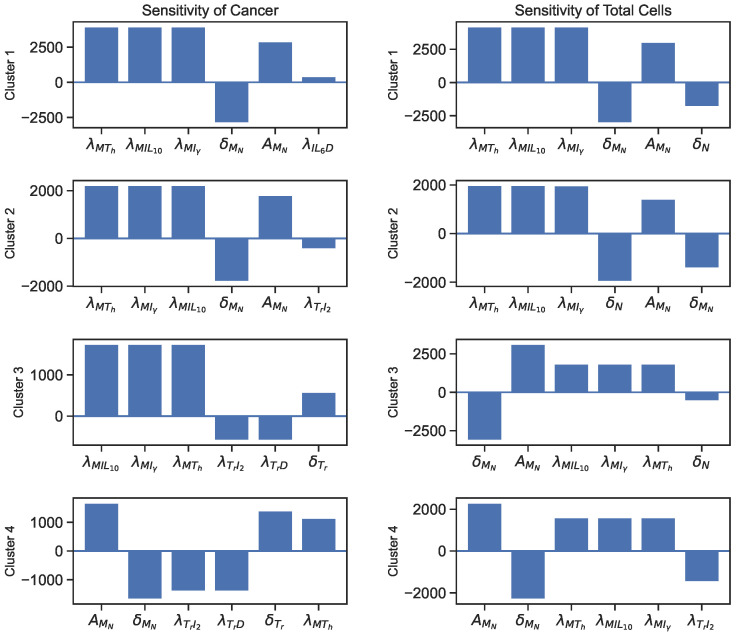
More sensitivity analysis results for cancer and total cells.

The sensitivity analysis reveals that the parameters directly involved in the cancer ODE are the most sensitive parameters for cancer cells and total cells. Additionally, macrophage-related parameters, such as $\lambda_{MT_{h}}$, $\lambda_{MIL_{10}}$, and $\lambda_{MI_{\gamma }}$, and T-reg cell-related parameters, such as $\lambda_{T_{r}D}$ and $\lambda_{T_{r}I_{2}}$, and an IL-6-related parameter, $\lambda_{IL_{6}D}$, also show significant sensitivity values. The sensitivity plots for the most varying parameters on cancer cells and total cells are shown in Figure 4 and Figure 5.

We could say that a quantity is positively sensitive to a parameter when an increase in the parameter causes the quantity to become larger. A quantity is negatively sensitive to a parameter when an increase in the parameter causes a decrease in the quantity. According to the sensitivity plot in Figure 4, cancer cells in all clusters are negatively sensitive to their decay rates and then positively sensitive to their growth rates. For all clusters, the most sensitive parameters for total cells are the decay/inhibition rates of cancer and macrophages and cancer cell growth/promotion rates.

In addition, Figure 5 illustrates more sensitive parameters for cancer cells and total cells. For sensitivity to cancer and total cells, we see a significant emphasis on macrophage-related parameters in all clusters, especially clusters 1 and 2. Additionally, regulatory T-cell promotion and decay rates play more significant parts in the cancer sensitivity results for clusters 3 and 4. Finally, we see $\lambda_{IL_{6}D}$ as the sixth sensitive parameter for cancer in cluster 1.

The sensitivity analyses of the parameters show that the direct and indirect interactions between the variables impact cancer or total cell growth in the cancer microenvironment. For instance, the inhibition of cancer cells by cytotoxic T-cells, IFN-γ, or natural decay, and its promotion by $IL_{6}$ and natural growth, are direct pathways that increase or decrease the number of cancer cells. On the other hand, we notice that cancer cells are negatively sensitive to the decay rate of macrophages (Figure 4) and positively sensitive to their promotion rates (Figure 5). This is because the macrophages secrete IL-6, and then IL-6 helps promote cancer. So, decay in macrophages would lead to less secretion of IL-6, leaving cancer cells with fewer resources and vice versa. Moreover, T-reg cell parameters play an important role in cancer development by negatively impacting cancer with their growth and positively affecting cancer with their decay, especially in clusters 3 and 4. Let us only consider that T-reg cells inhibit helper-T cells and cytotoxic cells. This correlation does not seem reasonable as helper T-cells promote cytotoxic cells and cytotoxic cells kill cancer cells. However, T-reg cells secrete HMGB1, which promotes dendritic cells, and both HMGB1 and dendritic cells promote helper T-cells, which help promote cytotoxic cells that kill cancer cells. Thus, through this pathway, T-reg cells negatively impact cancer cells with their growth and positively impact cancer cells with their decay rate.

### 3.3. Varying Dynamics of Cancer Cells with Scaled Assumptions

Appendix A.1 shows that most of the parameters (except possibly some decay rates), including the sensitive ones, were derived from restrictive assumptions. To assess the validity of these assumptions, we scaled them using factors of 1, 0.2, and 5. These scalings created a new set of parameters with significantly different values. Then from these sets, we perturbed the most sensitive parameters illustrated in Figure 4 and Figure 5 by 5% to create an interval of confidence for the new dynamics. After scaling all the assumptions in Appendix A.1, only 3 caused significant changes to cancer dynamics. These assumptions are as follows.

(20) $$\begin{array}{cc} \; & \mathrm{Scale}\times \delta_{DC}\left[C^{\max}\right]=50\delta_{D}, \end{array}$$

(21) $$\begin{array}{cc} \; & \mathrm{Scale}\times \lambda_{IL_{6}D}\left[D^{\max}\right]=\frac{2}{3}\lambda_{IL_{6}C}\left[C^{\max}\right]. \end{array}$$

(22) $$\begin{array}{cc} \; & \mathrm{Scale}\times \lambda_{IL_{6}T_{h}}\left[T_{h}^{\max}\right]=\frac{2}{3}\lambda_{IL_{6}C}\left[C^{\max}\right], \end{array}$$

**Figure 6:**
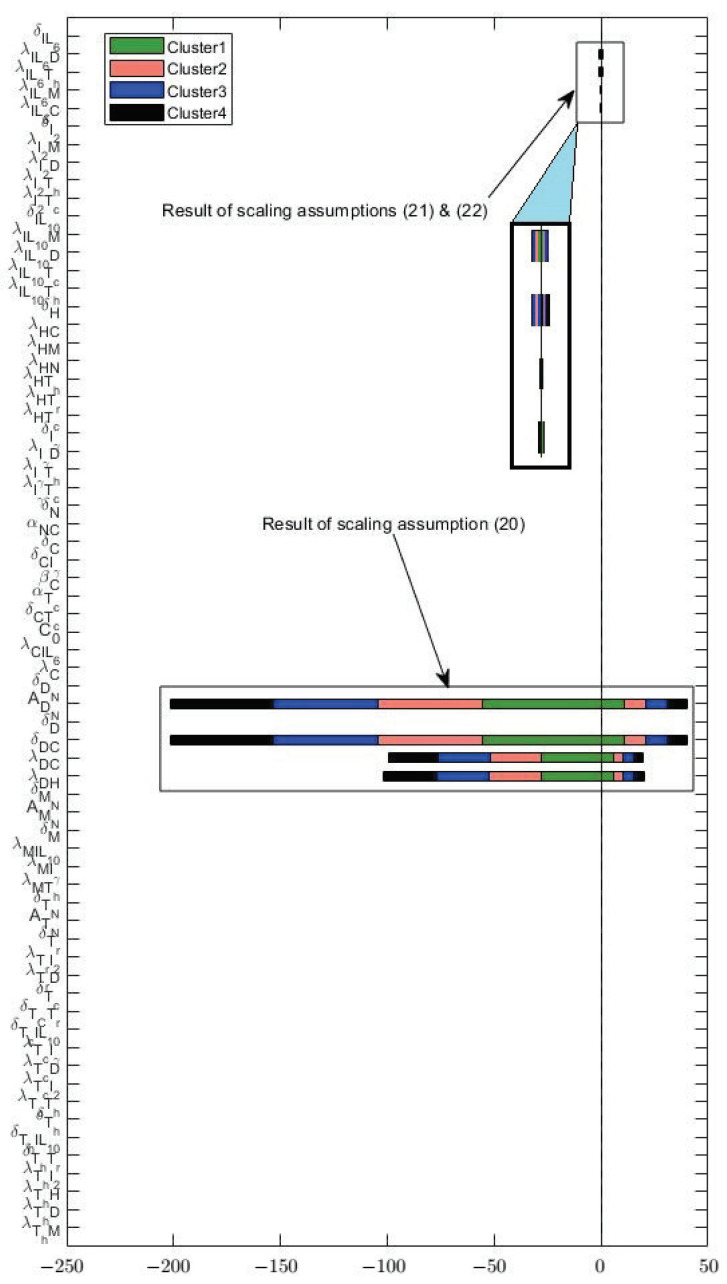
Deviations in parameter values from their baseline due to scaling assumptions (20)–(22).

**Figure 7:**
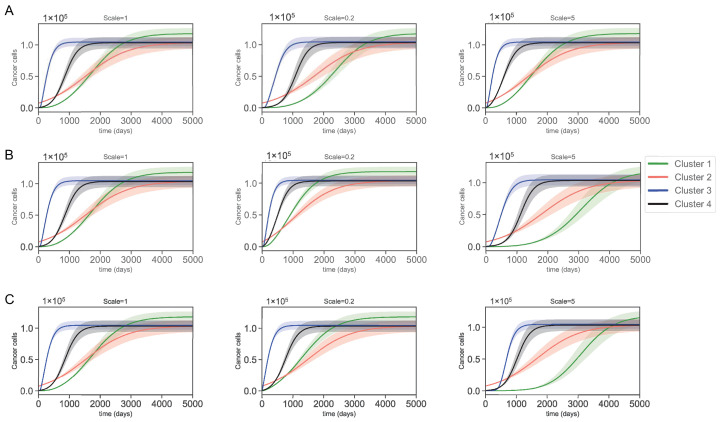
Cancer cell population after using scales 1, 0.2, and 5 in (**A**) assumption (20), (**B**) assumption (21), and (**C**) assumption (22) with the transparent region corresponding to 5% perturbation of all of the most sensitive parameters.

These three scalings cause significant changes in the parameters as illustrated in Figure 6. We can see that assumption (20) causes a significant deviation from the original values for parameters $A_{D_{N}}$, $\delta_{DC}$, $\lambda_{DC}$, and $\lambda_{DH}$. Moreover, scaling assumptions (21) and (22) causes parameters, such as $\lambda_{IL_{6}D}$, $\lambda_{IL_{6}T_{h}}$, $\lambda_{IL_{6}M}$, and $\lambda_{IL_{6}C}$ to change but not as drastically as the changes imposed by assumption (20). What is interesting is that none of these parameters (except for $\lambda_{IL_{6}D}$ in cluster 1) are among the sensitive parameters. However, the changes caused by the scaling of the assumptions were so large that the effects were tangible. We emphasize that none of the other assumptions left such an impact after scaling. Figure 7 shows the cancer dynamics with the original parameter values next to the dynamics acquired by scaling the assumptions (20)–(22) by 0.2 and 5, respectively. The shaded regions are the regions of confidence acquired from perturbing the most sensitive parameters in Figure 4 and Figure 5 by 5%. We see slight changes in clusters 2, 3, and 4, and a more significant change in cluster 1. Moreover, the width of the shaded regions remained the same in all cases. We saw that by changing the assumptions (20)–(22), significant deviations occurred to the parameter values mainly involved in the dendritic cells and $IL_{6}$ production. Cluster 1, which was more impacted, was the only cluster sensitive to the parameter $\lambda_{IL_{6}D}$. Even though the assumptions are mostly modeling artifacts for parameter estimations (and one must be cautious when using them), these results suggest interesting control potentials for $IL_{6}$.

**Figure 8:**
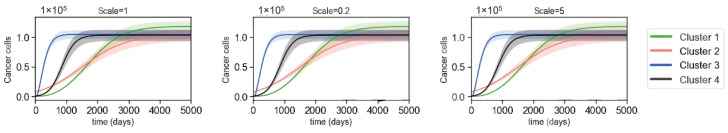
Cancer cell dynamics after scaling $\alpha_{T_{c}}$ with 1, 0.2, and 5 from left to right. The transparent regions are acquired from 5% perturbation of the most sensitive parameters.

Although the parameters $\alpha_{T_{c}}$ and $\beta_{C}$ were not among the most sensitive parameters, to make sure that the assumptions on these parameters were reasonable, we scaled $\alpha_{T_{c}}$ with 0.2 and 5 with 5% variations to the most sensitive parameters to see their impacts on cancer cells (Figure 8). Since $\alpha_{T_{c}}$ is multiplied by $\beta_{C}$ in the ODE system, we did not scale it as our goal was to keep the scaling of the term that involved these parameters similar to $\alpha_{T_{c}}$. Thus, the scaled equation for $\alpha_{T_{C}}$ becomes,

$$\mathrm{Scale}\times \alpha_{T_{c}}=8.9\cdot 10^{-5}\left[C^{\max}\right].$$

As a result, the term involving $\alpha_{T_{C}}$ in the cancer cell equation becomes,

$$\mathrm{Scale}\times \alpha_{T_{C}}\left[T_{C}\right]\beta_{C}\left[C\right].$$

The figure indicates that the dynamic of the cancer cell population remains unchanged after varying the term $\alpha_{T_{C}}\left[T_{C}\right]\beta_{C}\left[C\right]$. Moreover, the perturbation of the sensitive parameters causes similar variations in all three cases.

## 4. Discussion

Cancer is a diverse and complex disease that cannot be understood with a unified approach. Each cancer patient is unique in his/her immune infiltration profile and cancer type. In this paper, we proposed a model describing the critical interactions in the ccRCC tumor microenvironment. The model was inspired by the emerging clinical model of targeted therapy efficacy within distinct ccRCC subgroups. These models consider the infiltrating immune cells, such as cytotoxic, helper, and regulatory T-cells, macrophages, and dendritic cells (see Figure 1 in [36]). Therefore, we clustered four immunologically different virtual patients and observed the development of their tumors through their tumor microenvironment interactions. One of the main challenges in this study is the shortage of biological data for estimating the parameters in the model. So we added some mathematical (yet biologically reasonable) steady state assumptions to circumvent this issue. We then assessed the validity of these assumptions and the acquired parameter values in two ways. (1) By performing a global sensitivity analysis and observing the effects of varying the most sensitive parameters on cancer dynamics, and (2) by scaling our assumptions and studying their effects on cancer dynamics.

Despite the limitations of the model, we could infer interesting pathways in ccRCC and its microenvironment development. For instance, pathways that directly or indirectly affect the cytotoxicity of the microenvironment seemed to affect the cancer dynamics. We saw that a higher population of cytotoxic cells in cluster 2 is correlated to slower cancer cell growth. This could indicate that environments with initially higher cytotoxicity have a better shot at controlling ccRCC. Furthermore, the slow cancer growth in cluster 1 at the beginning can be related to naive dendritic cell growth patterns. More naive dendritic cells result in more mature dendritic cells that promote helper T-cells, aiding in more cytotoxicity in the environment [68]. Although cytotoxic cells were higher in population cluster 4, macrophages started to deplete soon for this cohort of patients, which caused faster growth in cancer cells via helper T-cell and cytotoxic cell pathways. Similar behavior can be justified for cluster 3. Another important relationship we observed in cluster 3 was that the dynamics of dendritic cells, INF-γ, IL-10, IL-6, and IL-2 and IL-12 looked very similar. Dendritic cells secreted all of the cytokines mentioned above, indicating that they played an important role in leading the dynamics of these cytokines in cluster 3. While increased IFN-γ may inhibit cancer cells or IL-2 and IL-12 may promote cytotoxicity, the inhibition of cytotoxic cells and helper T-cells by IL-10 or the promotion of cancer cells by IL-6 may have impacted more in aiding cancer cells to grow very fast in cluster 3. Therefore, although cancer cells are not directly impacted by macrophages or dendritic cells, cancer cell interaction with the cells and cytokines that macrophages and dendritic cells promote reveal potential pathways for better understanding the ccRCC microenvironment.

We performed a global sensitivity analysis on the parameters of the model to see their impacts on cancer and total cell growth. We saw that the most sensitive parameters were the production/promotion and decay/inhibition rates directly involved in cancer ODE, which was expected. In addition, we noticed a lot of macrophage-related production and inhibition rates as the second most sensitive parameters. Specifically, the sensitivity values hinted that more macrophages lead to worse prognoses. It has been shown that macrophage polarization into anti- and pro-tumor subtypes can have different implications for cancer progression and prognosis [77,78]. The patient data and the parameter sensitivity analysis of the model reveal that the macrophages act as pro-tumors in all patients, suggesting them as targets to suppress via therapy. Additionally, cytokines and cells act as promoters or inhibitors in sensitive parameters, such as IFN-γ, CD8+ T-cells, $IL_{6}$, $IL_{10}$, regulatory T-cells, dendritic cells, $IL_{2}$, and $IL_{12}$ can be potential targets. We also assessed the validity of our assumptions by scaling them. Almost all the assumptions led to no significant changes in the dynamics of cancer cells, except for three. These three assumptions, directly or indirectly, contributed to the significant production or inhibition of $IL_{6}$. Numerous studies focus on controlling the IL-6 as a therapeutic option to control the progression of ccRCC [79,80,81,82].

As with all the other mathematical models, our model is not without limitations. Many assumptions were used here, even though biologically feasible, but they were of mere mathematical nature. Some examples of such assumptions are: (1) the estimation of initial conditions by the small tumors in each cluster and assuming the largest tumors to give us the steady state values, and (2) the specific scales used to enforce the dominant activator or inhibitor of certain cells or cytokines. We tried our best to assess the sensitivity of our model to these assumptions and justify their robustness. However, none of these attempts can replace a direct validation using time-course data, and we acknowledge this limitation of the study. Moreover, for simplicity, we ignored important factors, such as angiogenesis and metabolites. We acknowledge this as another limitation of our model. However, this model provides a basis for future computational models of the ccRCC tumor microenvironment and can be extended to include new biological and biomedical discoveries and find optimal treatment options for ccRCC patients.

## Figures and Tables

**Table 1:** Variables and their correspondence to data.

Variables	Names	Combinations from Data
$T_{h}$	Helper T-cells	Activated memory CD4 T-cells and follicular helper T-cells
$T_{c}$	Cytotoxic cells	CD8 T-cells and activated NK cells
$T_{r}$	Regulatory T-cells	Regulatory T-cells
$T_{N}$	Naive T-cells	Naive CD4 T-cells, memory resting CD4 T-cells
		and resting natural killer (NK) cells
$D_{N}$	Naive dendritic cells	Naive dendritic cells
*D*	Dendritic cells	Mature dendritic cells
$M_{N}$	Naive Macrophages	M0 macrophages and monocytes
*M*	Macrophages	M1 and M2 macrophages
*C*	Cancer cells	Estimated from the data
*N*	Necrotic cells	Estimated from the data
$I_{\gamma }$	IFN-γ	Interferon-γ from gene expression data
*H*	HMGB1	HMGB1 from gene expression data
$IL_{10}$	IL-10	IL10 from gene expression data
$I_{2}$	IL-2 and IL-12	IL2 and IL12 from gene expression data
$IL_{6}$	IL-6	IL6 from gene expression data

**Table 2:** Nondimensionalized initial conditions of each cluster.

Cells and Cytokines	Cluster 1	Cluster 2	Cluster 3	Cluster 4
$\left[T_{h}\right]$	6.41	1.33	$3.24\cdot 10^{-1}$	$4.40\cdot 10^{-1}$
$\left[T_{C}\right]$	$9.99\cdot 10^{-1}$	$9.90\cdot 10^{-1}$	2.33	$8.65\cdot 10^{-1}$
$\left[T_{r}\right]$	$4.66\cdot 10^{-1}$	$8.80\cdot 10^{-1}$	1.42	$7.53\cdot 10^{-2}$
$\left[T_{N}\right]$	1.33	$1.35\cdot 10^{-3}$	$8.21\cdot 10^{-1}$	2.71
[M]	1.04	2.20	1.29	1.42
$\left[M_{N}\right]$	$8.26\cdot 10^{-1}$	1.21	$6.89\cdot 10^{-1}$	$2.62\cdot 10^{-1}$
[D]	$4.27\cdot 10^{1}$	1	$4.65\cdot 10^{-2}$	$1.56\cdot 10^{1}$
$\left[D_{N}\right]$	$5.98\cdot 10^{-1}$	$1.97\cdot 10^{1}$	$5.17\cdot 10^{1}$	1
[C]	$6.19\cdot 10^{-4}$	$7.20\cdot 10^{-2}$	$9.57\cdot 10^{-5}$	$4.92\cdot 10^{-3}$
[N]	$6.19\cdot 10^{-4}$	$7.20\cdot 10^{-2}$	$5.74\cdot 10^{-4}$	$4.92\cdot 10^{-3}$
$\left[I_{\gamma }\right]$	$4.82\cdot 10^{-1}$	2.17	1.06	$2.11\cdot 10^{-1}$
[H]	$8.95\cdot 10^{-1}$	1.15	$9.91\cdot 10^{-1}$	$9.12\cdot 10^{-1}$
$\left[IL_{10}\right]$	$2.31\cdot 10^{-1}$	6.03	1.28	$4.31\cdot 10^{-1}$
$\left[I_{2}\right]$	$4.95\cdot 10^{-1}$	2.69	$5.88\cdot 10^{-1}$	$7.78\cdot 10^{-1}$
$\left[IL_{6}\right]$	$4.08\cdot 10^{-1}$	$8.82\cdot 10^{-1}$	$2.45\cdot 10^{-1}$	$9.15\cdot 10^{-1}$

**Table 3:** Steady state values of the cells and cytokines.

Cells and Cytokines	Cluster 1	Cluster 2	Cluster 3	Cluster 4
$\left[T_{h}\right]$	$2.36\cdot 10^{2}$	$6.85\cdot 10^{2}$	$1.44\cdot 10^{3}$	$4.85\cdot 10^{3}$
$\left[T_{c}\right]$	$1.12\cdot 10^{4}$	$2.50\cdot 10^{4}$	$3.99\cdot 10^{3}$	$1.93\cdot 10^{4}$
$\left[T_{r}\right]$	$2.52\cdot 10^{3}$	$2.73\cdot 10^{3}$	$1.17\cdot 10^{3}$	$2.50\cdot 10^{3}$
$\left[T_{N}\right]$	$1.31\cdot 10^{4}$	$7.41\cdot 10^{3}$	$8.80\cdot 10^{3}$	$3.65\cdot 10^{3}$
[M]	$1.25\cdot 10^{4}$	$6.57\cdot 10^{3}$	$1.47\cdot 10^{4}$	$1.16\cdot 10^{4}$
$\left[M_{N}\right]$	$1.42\cdot 10^{3}$	$3.21\cdot 10^{3}$	$1.39\cdot 10^{4}$	$5.05\cdot 10^{3}$
[D]	$1.00\cdot 10^{1}$	$1.00\cdot 10^{1}$	$2.15\cdot 10^{2}$	$1.00\cdot 10^{1}$
$\left[D_{N}\right]$	$3.38\cdot 10^{3}$	$1.00\cdot 10^{1}$	$3.06\cdot 10^{1}$	$1.00\cdot 10^{1}$
[C]	$1.18\cdot 10^{5}$	$1.04\cdot 10^{5}$	$1.05\cdot 10^{5}$	$1.03\cdot 10^{5}$
[N]	$1.97\cdot 10^{4}$	$1.73\cdot 10^{4}$	$1.74\cdot 10^{4}$	$1.72\cdot 10^{4}$
$\left[I_{\gamma }\right]$	$3.85\cdot 10^{-2}$	$6.12\cdot 10^{-2}$	$1.92\cdot 10^{-2}$	$1.50\cdot 10^{-1}$
[H]	$3.63\cdot 10^{-1}$	$3.25\cdot 10^{-1}$	$3.28\cdot 10^{-1}$	$3.34\cdot 10^{-1}$
$\left[IL_{10}\right]$	$4.13\cdot 10^{-2}$	$1.24\cdot 10^{-2}$	$1.02\cdot 10^{-1}$	$6.84\cdot 10^{-2}$
$\left[I_{2}\right]$	$5.14\cdot 10^{-2}$	$1.36\cdot 10^{-2}$	$3.72\cdot 10^{-2}$	$4.86\cdot 10^{-2}$
$\left[IL_{6}\right]$	$6.07\cdot 10^{-2}$	$2.74\cdot 10^{-2}$	$2.74\cdot 10^{-1}$	$2.54\cdot 10^{-1}$

## Data Availability

All of the codes and data are available at https://github.com/ShahriyariLab/Patient-Specific-Mathematical-Model-of-Clear-Cell-Renal-Cell-Carcinoma-Microenvironme (accessed on 8 August 2022).

## Abbreviations

The following abbreviations are used in this manuscript:

ccRCC	clear cell renal cell carcinoma
ODE	ordinary differential equation
NK	natural killer
PD	programmed cell death
PD-L	programmed cell death ligand
IFN-γ	interferon-γ
DAMP	damage-associated molecular pattern
HMGB1	high mobility group box-1
VEGF	vascular endothelial growth factor
IL	interleukin
TCGA	The Cancer Genome Atlas
TCN	total cell number
TIC	total immune cell

## Appendix A. Derivation of Sample Parameters

### Appendix A.1. Parameter Assumptions Based on Steady States

The average doubling time for renal cell carcinoma is 460.01 ± 182.45 days [83]. Thus, we considered the doubling rate to be the difference between the growth and decay rate of cancer cells, as was done in [16,17,18]. The faster doubling rate includes the rate of cancer cells’ natural proliferation and proliferation by IL-6, resulting in

(A1) $$\begin{array}{c} \frac{\ln2}{278}=\left(\lambda_{C}+\lambda_{IL_{6}}\left[CIL_{6}^{\mathrm{mean}}\right]\right)-\delta_{C}. \end{array}$$

Then, we consider the slower doubling rate to be only the natural cancer cell proliferation while the decay rates of cancer cells include the decay rates by all the inhibitors, including cancer’s natural decay rate,

(A2) $$\begin{array}{c} \frac{\ln2}{642}=\lambda_{C}-\left(\delta_{CT_{c}}\left[T_{c}^{\mathrm{mean}}\right]\cdot \left(\frac{1}{1+\alpha_{T_{c}}\left[T_{c}^{\mathrm{mean}}\right]\cdot \beta_{C}\left[C^{\mathrm{mean}}\right]}\right)+\delta_{CI_{\gamma }}\left[I_{\gamma }^{\mathrm{mean}}\right]+\delta_{C}\right). \end{array}$$

The mean values of the variables used in Equations (A1) and (A2) are given in Table A1.

**Table A1:** Mean values of the variables from the data.

$\left[{\mathrm{IL}}_{6}^{\mathrm{mean}}\right]$	$\left[T_{c}^{\mathrm{mean}}\right]$	$\left[C^{\mathrm{mean}}\right]$	$\left[I_{\gamma }^{\mathrm{mean}}\right]$
$9.751\cdot 10^{-2}$	$1.363\cdot 10^{4}$	$4.50\cdot 10^{4}$	$5.004\cdot 10^{-2}$

Since we assume the parameter relations based on the steady states of each variable, the parameters should all be positive.

The steady states are derived from the largest tumor across all patients. See Table A2.

**Table A2:** Steady state values of the variables derived from the largest tumors from each cluster.

$\left[T_{h}^{\max}\right]$	$\left[T_{c}^{\max}\right]$	$\left[T_{r}^{\max}\right]$	$\left[T_{n}^{\max}\right]$	$\left[M^{\max}\right]$
$2.360\cdot 10^{2}$	$1.119\cdot 10^{4}$	$2.522\cdot 10^{3}$	$1.306\cdot 10^{4}$	$1.245\cdot 10^{4}$
** $\left[M_{N}^{\max}\right]$ **	** $\left[D^{\max}\right]$ **	** $\left[D_{N}^{\max}\right]$ **	** $\left[C^{\max}\right]$ **	** $\left[N^{\max}\right]$ **
$1.425\cdot 10^{3}$	10	$3.384\cdot 10^{3}$	$1.182\cdot 10^{5}$	$1.971\cdot 10^{4}$
** $\left[I_{\gamma }^{\max}\right]$ **	** $\left[H^{\max}\right]$ **	** $\left[{\mathrm{IL}}_{10}^{\max}\right]$ **	** $\left[I_{2}^{\max}\right]$ **	** $\left[{\mathrm{IL}}_{6}^{\max}\right]$ **
$3.853\cdot 10^{-2}$	$3.630\cdot 10^{-1}$	$4.132\cdot 10^{-2}$	$5.143\cdot 10^{-2}$	$6.071\cdot 10^{-2}$

We assume that helper T-cells are mainly activated by macrophages and dendritic cells,

(A3) $$\begin{array}{c} \lambda_{T_{h}M}\left[M^{\max}\right]=\lambda_{T_{h}D}\left[D^{\max}\right]=200\lambda_{T_{h}H}\left[H^{\max}\right]=25\lambda_{T_{h}I_{2}}\left[I_{2}^{\max}\right]. \end{array}$$

Moreover, for helper T-cells, we assume that the natural inhibition is smaller than inhibition by T-reg cells and IL-10, so that

(A4) $$\begin{array}{c} \delta_{T_{h}Tr}\left[T_{r}^{\max}\right]=\delta_{T_{h}IL_{10}}\left[IL_{10}^{\max}\right]=20\delta_{T_{h}} \end{array}$$

For cytotoxic cells, we assume that the activation or proliferation is mainly done by helper T-cells, dendritic cells, and IL-2 since IL-2 has been described as an effective media to treat cancer [28,76], and we assume that the inhibitions are mainly done by T-reg cells and IL-10.

(A5) $$\begin{array}{cc} \; & \lambda_{T_{c}T_{h}}\left[T_{h}^{\max}\right]=\lambda_{T_{c}I_{2}}\left[I_{2}^{\max}\right]=\lambda_{T_{c}D}\left[D^{\max}\right]=100\lambda_{T_{c}I_{\gamma }}\left[I_{\gamma }^{\max}\right] \end{array}$$

(A6) $$\begin{array}{cc} \; & \delta_{T_{c}IL_{10}}\left[IL_{10}^{\max}\right]=\delta_{T_{c}T_{r}}\left[T_{r}^{\max}\right]=20\delta_{T_{c}} \end{array}$$

For T-reg cells, we assume that dendritic cells mainly activate them,

(A7) $$\begin{array}{c} \lambda_{T_{r}D}\left[D^{\max}\right]=100\lambda_{T_{r}I_{2}}\left[I_{2}^{\max}\right]. \end{array}$$

For macrophages, we assume proliferation scales of helper T-cells, IFN-γ, and IL-10 are similar, so we have,

(A8) $$\begin{array}{c} \lambda_{MT_{h}}\left[T_{h}^{\max}\right]=\lambda_{MI_{\gamma }}\left[I_{\gamma }^{\max}\right]=\lambda_{MIL_{10}}\left[IL_{10}^{\max}\right]. \end{array}$$

We further assume that the decay rates of naive macrophages are similar to the decay rates of macrophages,

(A9) $$\begin{array}{c} \delta_{M}=\delta_{M_{N}}. \end{array}$$

Similarly, we assume that the proliferation rates for dendritic cells are similar, and their inhibition rate by cancer cells is larger than their natural decay rate. Moreover, we take the decay rate of naive dendritic cells to be the same as that of dendritic cells.

(A10) $$\begin{array}{cc} \; & \lambda_{DH}\left[H^{\max}\right]=\lambda_{DC}\left[C^{\max}\right], \end{array}$$

(A11) $$\begin{array}{cc} \; & \delta_{DC}\left[C^{\max}\right]=50\delta_{D}, \end{array}$$

(A12) $$\begin{array}{cc} \; & \delta_{D_{N}}=\delta_{D}. \end{array}$$

We calculate the parameter $\alpha_{T_{c}}$ based on the average fraction of PD−1 and PD−2 among all the genes and then multiply it by the maximum of cytotoxic cells. Similarly, we calculate $\beta_{C}$ based on the average of PDL−2 among all genes(as it was the only available ligand in the data) and then multiply it with the cancer cell maximum. Since cancer cells grow rapidly, we let

(A13) $$\begin{array}{ccc} \; & C_{0}=2.5\left[C^{\max}\right],\; & \;\;\;\;\alpha_{T_{c}}=8.9\cdot 10^{-5}\left[T_{c}^{\max}\right], \end{array}$$

(A14) $$\begin{array}{ccc} & \;\beta_{C}=3.3\cdot 10^{-5}\left[C^{\max}\right],\; & \delta_{CI_{\gamma }}\left[I_{\gamma }^{\mathrm{mean}}\right]=\delta_{CT_{c}}\left[T_{C}^{\mathrm{mean}}\right] \end{array}$$

(A15) $$\begin{array}{ccc} \; & \;\;\mathrm{and}\;\; & \;\;\;\;\;\;\;\;\;\delta_{C}=\delta_{CT_{C}}\left[T_{C}^{\mathrm{mean}}\right]. \end{array}$$

For necrotic cells, we assume that

(A16) $$\begin{array}{c} \alpha_{NC}=0.5. \end{array}$$

We assume that IFN-γ is secreted mostly by cytotoxic cells,

(A17) $$\begin{array}{c} \lambda_{I_{\gamma }T_{c}}\left[T_{c}^{\max}\right]=5\lambda_{I_{\gamma }T_{h}}\left[T_{h}^{\max}\right]=\lambda_{I_{\gamma }D}\left[D^{\max}\right]. \end{array}$$

Since HMGB1 is mainly produced by cancer cells and necrotic cells, we let the HMGB1 production rate by cancer and necrotic be larger,

(A18) $$\begin{array}{cc} 10\lambda_{HT_{c}}\left[T_{c}^{\max}\right] & =10\lambda_{HT_{r}}\left[T_{r}^{\max}\right]=10\lambda_{HT_{h}}\left[T_{h}^{\max}\right]=10\lambda_{HM}\left[M^{\max}\right]\\ & =\lambda_{HN}\left[N^{\max}\right]=\lambda_{HC}\left[C^{\max}\right]. \end{array}$$

For IL-10, we assume that it is secreted on a similar scale by helper T-cells, T-reg cells, cytotoxic cells, dendritic cells, and macrophages;

(A19) $$\begin{array}{cc} \lambda_{IL_{10}T_{c}}\left[T_{c}^{\max}\right]\; & =\lambda_{IL_{10}T_{h}}\left[T_{h}^{\max}\right]=\lambda_{IL_{10}D}\left[D^{\max}\right] \end{array}$$

(A20) $$\begin{array}{cc} & =\lambda_{IL_{10}M}\left[M^{\max}\right]. \end{array}$$

Since helper T-cells mainly produce IL-2, we let the rate of IL-2 secretion by helper T-cells be larger [28]. We then let other cell production rates of IL-12 be similar.

(A21) $$\begin{array}{c} 2\lambda_{I_{2}T_{c}}\left[T_{c}^{\max}\right]=\lambda_{I_{2}T_{h}}\left[T_{h}^{\max}\right]=2\lambda_{I_{2}M}\left[M^{\max}\right]. \end{array}$$

Lastly, since IL-6 is mainly produced by cancer cells, we let

(A22) $$\begin{array}{c} \lambda_{IL_{6}C}\left[C^{\max}\right]=1.5\lambda_{IL_{6}M}\left[M^{\max}\right]=1.5\lambda_{IL_{6}T_{h}}\left[T_{h}^{\max}\right]=1.5\lambda_{IL_{6}D}\left[D^{\max}\right]. \end{array}$$

### Appendix A.2. Nondimensionalization of Parameters and Variables

First, consider the variables at a steady state as,

$$T_{h}^{\infty },T_{c}^{\infty },T_{r}^{\infty },T_{N}^{\infty },M^{\infty },M_{N}^{\infty },D^{\infty },D_{N}^{\infty },C^{\infty },N^{\infty },I_{\gamma }^{\infty },H^{\infty },IL_{10}^{\infty },I_{2}^{\infty },\;\mathrm{and}\;IL_{6}^{\infty }.$$

To avoid complexity in parameter estimations, numerically solving the ODEs, and analyzing the sensitivity of parameters, we nondimensionalize the variables and parameters based on their steady state values. We denote the nondimensional variables by $\left[\bar{X}\right]=\frac{\left[X\right]}{\left[X^{\infty }\right]}$, where *X* represents each variable and $[\bar{X}]=1$ when it is in a steady state. However, we do not nondimensionalize the time variable since it does not introduce any complexity to the problem. Thus, the system consisting of Equations (1)–(16) (except (9)) would be nondimensionalized, as follows:

(A23) $$\begin{array}{cc} \frac{d[\bar{T_{h}}]}{dt} & =\left(\bar{\lambda_{T_{h}M}}\left[\bar{M}\right]+\bar{\lambda_{T_{h}D}}\left[\bar{D}\right]+\bar{\lambda_{T_{h}H}}\left[\bar{H}\right]+\bar{\lambda_{T_{h}I_{2}}}\left[\bar{I_{2}}\right]\right)\left[\bar{T_{N}}\right]\\ & -\left(\bar{\delta_{T_{h}T_{r}}}\left[\bar{T_{r}}\right]+\bar{\delta_{T_{h}IL_{10}}}\left[\bar{IL_{10}}\right]+\delta_{T_{h}}\right)\left[\bar{T_{h}}\right] \end{array}$$

(A24) $$\begin{array}{cc} \frac{d[\bar{T_{c}}]}{dt} & =\left(\bar{\lambda_{T_{c}T_{h}}}\left[\bar{T_{h}}\right]+\bar{\lambda_{T_{c}I_{2}}}\left[\bar{I_{2}}\right]+\bar{\lambda_{T_{c}D}}\left[\bar{D}\right]+\bar{\lambda_{T_{c}I_{\gamma }}}\left[\bar{I_{\gamma }}\right]\right)\left[\bar{T_{N}}\right]\\ & -\left(\bar{\delta_{T_{c}IL_{10}}}\left[\bar{IL_{10}}\right]+\bar{\delta_{T_{c}T_{r}}}\left[\bar{T_{r}}\right]+\delta_{T_{c}}\right)\left[\bar{T_{c}}\right] \end{array}$$

(A25) $$\begin{array}{cc} \frac{d[\bar{T_{r}}]}{dt} & =\left(\bar{\lambda_{T_{r}D}}\left[\bar{D}\right]+\bar{\lambda_{T_{r}I_{2}}}\left[\bar{I_{2}}\right]\right)\left[T_{N}\right]-\delta_{T_{r}}\left[\bar{T_{r}}\right]. \end{array}$$

(A26) $$\begin{array}{cc} \frac{d[\bar{T_{N}}]}{dt} & =\bar{A_{T_{N}}}-\left(\bar{\lambda_{T_{h}M}}\left[\bar{M}\right]+\bar{\lambda_{T_{h}D}}\left[\bar{D}\right]+\bar{\lambda_{T_{h}H}}\left[\bar{H}\right]+\bar{\lambda_{T_{h}I_{2}}}\left[\bar{I_{2}}\right]\right)\left[\bar{T_{N}}\right]\\ & -\left(\bar{\lambda_{T_{c}T_{h}}}\left[\bar{T_{h}}\right]+\bar{\lambda_{T_{c}I_{2}}}\left[\bar{I_{2}}\right]+\bar{\lambda_{T_{c}D}}\left[\bar{D}\right]+\bar{\lambda_{T_{c}I_{\gamma }}}\left[\bar{I_{\gamma }}\right]\right)\left[\bar{T_{N}}\right]\\ & -\left(\bar{\lambda_{T_{r}D}}\left[\bar{D}\right]+\bar{\lambda_{T_{r}I_{2}}}\left[\bar{I_{2}}\right]+\delta_{T_{N}}\right)\left[\bar{T_{N}}\right] \end{array}$$

(A27) $$\begin{array}{cc} \frac{d[\bar{M}]}{dt} & =\left(\bar{\lambda_{MT_{h}}}\left[\bar{T_{h}}\right]+\bar{\lambda_{MI_{\gamma }}}\left[\bar{I_{\gamma }}\right]+\bar{\lambda_{MIL_{10}}}\left[\bar{IL_{10}}\right]\right)\left[\bar{M_{N}}\right]-\delta_{M}\left[\bar{M}\right] \end{array}$$

(A28) $$\begin{array}{cc} \frac{d[\bar{M_{N}}]}{dt} & =\bar{A_{M_{N}}}-\left(\bar{\lambda_{MT_{h}}}\left[\bar{T_{h}}\right]+\bar{\lambda_{MI_{\gamma }}}\left[\bar{I_{\gamma }}\right]+\bar{\lambda_{MIL_{10}}}\left[\bar{IL_{10}}\right]+\delta_{M_{N}}\right)\left[\bar{M_{N}}\right] \end{array}$$

(A29) $$\begin{array}{cc} \frac{d[\bar{D}]}{dt} & =\left(\bar{\lambda_{DH}}\left[\bar{H}\right]+\bar{\lambda_{DC}}\left[\bar{C}\right]\right)\left[\bar{D_{N}}\right]-\left(\bar{\delta_{DC}}\left[\bar{C}\right]+\delta_{D}\right)\left[\bar{D}\right] \end{array}$$

(A30) $$\begin{array}{cc} \frac{d[\bar{D_{N}}]}{dt} & =\bar{A_{D}}-\left(\bar{\lambda_{DH}}\left[\bar{H}\right]+\bar{\lambda_{DC}}\left[\bar{C}\right]+\delta_{D_{N}}\right)\left[\bar{D_{N}}\right] \end{array}$$

(A31) $$\begin{array}{cc} \frac{d[\bar{C}]}{dt} & =\left(\lambda_{C}+\bar{\lambda_{CIL_{6}}}\left[\bar{IL_{6}}\right]\right)\left(1-\frac{\left[\bar{C}\right]}{\bar{C_{0}}}\right)\left[\bar{C}\right]\\ & -\left(\bar{\delta_{CT_{c}}}\left[\bar{T_{c}}\right]\cdot \left(\frac{1}{1+\bar{\alpha_{T_{c}}}\left[\bar{T_{c}}\right]\cdot \bar{\beta_{C}}\left[\bar{C}\right]}\right)+\bar{\delta_{CI_{\gamma }}}\left[\bar{I_{\gamma }}\right]+\delta_{C}\right)\left[\bar{C}\right] \end{array}$$

(A32) $$\begin{array}{cc} \frac{d[\bar{N}]}{dt} & =\bar{\alpha_{NC}}\left(\bar{\delta_{CT_{c}}}\left[\bar{T_{c}}\right]\cdot \left(\frac{1}{1+\bar{\alpha_{T_{c}}}\left[\bar{T_{c}}\right]\cdot \bar{\beta_{C}}\left[\bar{C}\right]}\right)+\bar{\delta_{CI_{\gamma }}}\left[\bar{I_{\gamma }}\right]+\delta_{C}\right)\left[\bar{C}\right]-\delta_{N}\left[\bar{N}\right] \end{array}$$

(A33) $$\begin{array}{cc} \frac{d[\bar{I_{\gamma }}]}{dt} & =\bar{\lambda_{I_{\gamma }T_{c}}}\left[\bar{T_{c}}\right]+\bar{\lambda_{I_{\gamma }T_{h}}}\left[\bar{T_{h}}\right]+\bar{\lambda_{I_{\gamma }D}}\left[\bar{D}\right]-\delta_{I_{\gamma }}\left[\bar{I_{\gamma }}\right] \end{array}$$

(A34) $$\begin{array}{cc} \frac{d[\bar{H}]}{dt} & =\bar{\lambda_{HT_{c}}}\left[\bar{T_{c}}\right]+\bar{\lambda_{HT_{r}}}\left[\bar{T_{r}}\right]+\bar{\lambda_{HT_{h}}}\left[\bar{T_{h}}\right]+\bar{\lambda_{HN}}\left[\bar{N}\right]+\bar{\lambda_{HM}}\left[\bar{M}\right]+\bar{\lambda_{HC}}\left[\bar{C}\right]-\delta_{H}\left[\bar{H}\right] \end{array}$$

(A35) $$\begin{array}{cc} \frac{d[\bar{IL_{10}}]}{dt} & =\bar{\lambda_{IL_{10}T_{h}}}\left[\bar{T_{h}}\right]+\bar{\lambda_{IL_{10}T_{c}}}\left[\bar{T_{c}}\right]+\bar{\lambda_{IL_{10}D}}\left[\bar{D}\right]+\bar{\lambda_{IL_{10}M}}\left[\bar{M}\right]-\delta_{IL_{10}}\left[\bar{IL_{10}}\right] \end{array}$$

(A36) $$\begin{array}{cc} \frac{d[\bar{I_{2}}]}{dt} & =\bar{\lambda_{I_{2}T_{c}}}\left[\bar{T_{c}}\right]+\bar{\lambda_{I_{2}T_{h}}}\left[\bar{T_{h}}\right]+\bar{\lambda_{I_{2}D}}\left[\bar{D}\right]+\bar{\lambda_{I_{2}M}}\left[\bar{M}\right]-\delta_{I_{2}}\left[\bar{I_{2}}\right] \end{array}$$

(A37) $$\begin{array}{cc} \frac{d[\bar{IL_{6}}]}{dt} & =\bar{\lambda_{IL_{6}C}}\left[\bar{C}\right]+\bar{\lambda_{IL_{6}M}}\left[\bar{M}\right]+\bar{\lambda_{IL_{6}T_{h}}}\left[\bar{T_{h}}\right]+\bar{\lambda_{IL_{6}D}}\left[\bar{D}\right]-\delta_{IL_{6}}\left[\bar{IL_{6}}\right] \end{array}$$

Since the dimension of the time variable remains unchanged, the decay rates in Table A3 also remain unchanged. We nondimensionalize the assumptions in Appendix A.1 as follows:

(A38) $$\begin{array}{cc} \; & \frac{\ln2}{278}=\left(\lambda_{C}+\lambda_{IL_{6}}\frac{\left[IL_{6}^{\mathrm{mean}}\right]}{\left[IL_{6}^{\infty }\right]}\right)-\delta_{C}, \end{array}$$

(A39) $$\begin{array}{cc} \; & \frac{\ln2}{642}=\lambda_{C}-\left(\delta_{CT_{c}}\frac{\left[T_{c}^{\mathrm{mean}}\right]}{\left[T_{c}^{\infty }\right]}\cdot \left(\frac{1}{1+\alpha_{T_{c}}\frac{\left[T_{c}^{\mathrm{mean}}\right]}{\left[T_{c}^{\infty }\right]}\cdot \beta_{C}\frac{\left[C^{\mathrm{mean}}\right]}{\left[C^{\infty }\right]}}\right)+\delta_{CI_{\gamma }}\frac{\left[I_{\gamma }^{\mathrm{mean}}\right]}{\left[I_{\gamma }^{\infty }\right]}+\delta_{C}\right), \end{array}$$

(A40) $$\begin{array}{cc} \; & \lambda_{T_{h}M}\frac{\left[M^{\max}\right]}{\left[M^{\infty }\right]}=\lambda_{T_{h}D}\frac{\left[D^{\max}\right]}{\left[D^{\infty }\right]}=200\lambda_{T_{h}H}\frac{\left[H^{\max}\right]}{\left[H^{\infty }\right]}=25\lambda_{T_{h}I_{2}}\frac{\left[I_{2}^{\max}\right]}{\left[I_{2}^{\infty }\right]}, \end{array}$$

(A41) $$\begin{array}{cc} \; & \delta_{T_{h}Tr}\frac{\left[T_{r}^{\max}\right]}{\left[T_{r}^{\infty }\right]}=\delta_{T_{h}IL_{10}}\frac{\left[IL_{10}^{\max}\right]}{\left[IL_{10}^{\infty }\right]}=20\delta_{T_{h}}, \end{array}$$

(A42) $$\begin{array}{cc} \; & \lambda_{T_{c}T_{h}}\frac{\left[T_{h}^{\max}\right]}{\left[T_{h}^{\infty }\right]}=\lambda_{T_{c}I_{2}}\frac{\left[I_{2}^{\max}\right]}{\left[I_{2}^{\infty }\right]}=\lambda_{T_{c}D}\frac{\left[D^{\max}\right]}{\left[D^{\infty }\right]}=100\lambda_{T_{c}I_{\gamma }}\frac{\left[I_{\gamma }^{\max}\right]}{\left[I_{\gamma }^{\infty }\right]}, \end{array}$$

(A43) $$\begin{array}{cc} \; & \delta_{T_{c}IL_{10}}\frac{\left[IL_{10}^{\max}\right]}{\left[IL_{10}^{\infty }\right]}=\delta_{T_{c}T_{r}}\frac{\left[T_{r}^{\max}\right]}{\left[T_{r}^{\infty }\right]}=20\delta_{T_{c}}, \end{array}$$

(A44) $$\begin{array}{cc} \; & \lambda_{T_{r}D}\frac{\left[D^{\max}\right]}{\left[D^{\infty }\right]}=100\lambda_{T_{r}I_{2}}\frac{\left[I_{2}^{\max}\right]}{\left[I_{2}^{\infty }\right]}, \end{array}$$

(A45) $$\begin{array}{cc} \; & \lambda_{MT_{h}}\frac{\left[T_{h}^{\max}\right]}{\left[T_{h}^{\infty }\right]}=\lambda_{MI_{\gamma }}\frac{\left[I_{\gamma }^{\max}\right]}{\left[I_{\gamma }^{\infty }\right]}=\lambda_{MIL_{10}}\frac{\left[IL_{10}^{\max}\right]}{\left[IL_{10}^{\infty }\right]}, \end{array}$$

(A46) $$\begin{array}{cc} \; & \lambda_{DH}\frac{\left[H^{\max}\right]}{\left[H^{\infty }\right]}=\lambda_{DC}\frac{\left[C^{\max}\right]}{\left[C^{\infty }\right]}, \end{array}$$

(A47) $$\begin{array}{cc} \; & \delta_{DC}\frac{\left[C^{\max}\right]}{\left[C^{\infty }\right]}=50\delta_{D}, \end{array}$$

(A48) $$\begin{array}{cc} \; & \lambda_{I_{\gamma }T_{c}}\frac{\left[T_{c}^{\max}\right]}{\left[T_{c}^{\infty }\right]}=5\lambda_{I_{\gamma }T_{h}}\frac{\left[T_{h}^{\max}\right]}{\left[T_{h}^{\infty }\right]}=\lambda_{I_{\gamma }D}\frac{\left[D^{\max}\right]}{\left[D^{\infty }\right]}, \end{array}$$

(A49) $$\begin{array}{c} 10\lambda_{HT_{c}}\frac{\left[T_{c}^{\max}\right]}{\left[T_{c}^{\infty }\right]}=10\lambda_{HT_{r}}\frac{\left[T_{r}^{\max}\right]}{\left[T_{r}^{\infty }\right]}=10\lambda_{HT_{h}}\frac{\left[T_{h}^{\max}\right]}{\left[T_{h}^{\infty }\right]}=10\lambda_{HM}\frac{\left[M^{\max}\right]}{\left[M^{\infty }\right]}\\ =\lambda_{HN}\frac{\left[N^{\max}\right]}{\left[N^{\infty }\right]}=\lambda_{HC}\frac{\left[C^{\max}\right]}{\left[C^{\infty }\right]}, \end{array}$$

(A50) $$\begin{array}{cc} \; & \lambda_{IL_{10}T_{c}}\frac{\left[T_{c}^{\max}\right]}{\left[T_{c}^{\infty }\right]}=\lambda_{IL_{10}T_{h}}\frac{\left[T_{h}^{\max}\right]}{\left[T_{h}^{\infty }\right]}=\lambda_{IL_{10}D}\frac{\left[D^{\max}\right]}{\left[D^{\infty }\right]}=\lambda_{IL_{10}M}\frac{\left[M^{\max}\right]}{\left[M^{\infty }\right]}, \end{array}$$

(A51) $$\begin{array}{cc} \; & 2\lambda_{I_{2}T_{c}}\frac{\left[T_{c}^{\max}\right]}{\left[T_{c}^{\infty }\right]}=\lambda_{I_{2}T_{h}}\frac{\left[T_{h}^{\max}\right]}{\left[T_{h}^{\infty }\right]}=2\lambda_{I_{2}M}\frac{\left[M^{\max}\right]}{\left[M^{\infty }\right]}, \end{array}$$

(A52) $$\begin{array}{cc} \; & \lambda_{IL_{6}C}\frac{\left[C^{\max}\right]}{\left[C^{\infty }\right]}=1.5\lambda_{IL_{6}M}\frac{\left[M^{\max}\right]}{\left[M^{\infty }\right]}=1.5\lambda_{IL_{6}T_{h}}\frac{\left[T_{h}^{\max}\right]}{\left[T_{h}^{\infty }\right]}=1.5\lambda_{IL_{6}D}\frac{\left[D^{\max}\right]}{\left[D^{\infty }\right]}, \end{array}$$

(A53) $$\begin{array}{cc} \; & \bar{C_{0}}=2.5\frac{\left[C^{\max}\right]}{\left[C^{\infty }\right]},\;\bar{\alpha_{T_{c}}}=8.9\cdot 10^{-5}\frac{\left[T_{c}^{\max}\right]}{\left[T_{c}^{\infty }\right]}, \end{array}$$

(A54) $$\begin{array}{cc} & \bar{\beta_{C}}=3.3*10^{-5}\frac{\left[C^{\max}\right]}{\left[C^{\infty }\right]}, \end{array}$$

(A55) $$\begin{array}{cc} & \delta_{CI_{\gamma }}\frac{\left[I_{\gamma }^{\mathrm{mean}}\right]}{\left[I_{\gamma }^{\infty }\right]}=\delta_{CT_{c}}\frac{\left[T_{c}^{\mathrm{mean}}\right]}{\left[T_{c}^{\infty }\right]}, \end{array}$$

(A56) $$\begin{array}{cc} \; & \delta_{C}=\delta_{CT_{C}}\frac{\left[T_{c}^{\mathrm{mean}}\right]}{\left[T_{c}^{\infty }\right]}. \end{array}$$

In addition, we nondimensionalize independent production rates, such as $A_{T_{N}}$, $A_{M_{N}}$, and $A_{D}$ by $\frac{A_{T_{N}}}{\left[T_{N}^{\infty }\right]}$, $\frac{A_{M_{N}}}{\left[M_{N}^{\infty }\right]}$, and $\frac{A_{D}}{\left[D^{\infty }\right]}$, respectively. According to the above nondimensionalization of the system and assumptions, we have the nondimensional parameters in Table A3.

**Table A3:** Nondimensionalized parameters.

Parameter	Cluster 1	Cluster 2	Cluster 3	Cluster 4
$\bar{\lambda_{T_{h}M}}$	4.71	2.28	$7.16\cdot 10^{-1}$	5.98
$\bar{\lambda_{T_{h}D}}$	4.71	4.32	$1.30\cdot 10^{1}$	6.41
$\bar{\lambda_{T_{h}H}}$	$2.36\cdot 10^{-2}$	$1.93\cdot 10^{-2}$	$2.74\cdot 10^{-3}$	$2.95\cdot 10^{-2}$
$\bar{\lambda_{T_{h}I_{2}}}$	$2.36\cdot 10^{-2}$	$5.72\cdot 10^{-3}$	$2.19\cdot 10^{-3}$	$3.03\cdot 10^{-2}$
$\bar{\delta_{T_{h}T_{r}}}$	4.62	5.00	2.15	4.57
$\bar{\delta_{T_{h}IL_{10}}}$	4.62	1.38	$1.14\cdot 10^{1}$	7.65
$\delta_{T_{h}}$	$2.31\cdot 10^{-1}$	$2.31\cdot 10^{-1}$	$2.31\cdot 10^{-1}$	$2.31\cdot 10^{-1}$
$\bar{\lambda_{T_{c}T_{h}}}$	5.53	8.07	5.21	$2.00\cdot 10^{1}$
$\bar{\lambda_{T_{c}I_{2}}}$	5.53	$7.38\cdot 10^{-1}$	$6.16\cdot 10^{-1}$	$9.18\cdot 10^{-1}$
$\bar{\lambda_{T_{c}D}}$	5.53	2.78	$1.83\cdot 10^{1}$	$9.71\cdot 10^{-1}$
$\bar{\lambda_{T_{c}I_{\gamma }}}$	$5.53\cdot 10^{-2}$	$4.42\cdot 10^{-2}$	$4.25\cdot 10^{-3}$	$3.78\cdot 10^{-2}$
$\bar{\delta_{T_{c}IL_{10}}}$	8.12	2.43	$2.00\cdot 10^{1}$	$1.34\cdot 10^{1}$
$\bar{\delta_{T_{C}T_{r}}}$	8.12	8.79	3.78	8.04
$\delta_{T_{c}}$	$4.06\cdot 10^{-1}$	$4.06\cdot 10^{-1}$	$4.06\cdot 10^{-1}$	$4.06\cdot 10^{-1}$
$\bar{\lambda_{T_{r}D}}$	$2.29\cdot 10^{-1}$	$2.30\cdot 10^{-1}$	$2.31\cdot 10^{-1}$	$2.29\cdot 10^{-1}$
$\bar{\lambda_{T_{r}I_{2}}}$	$2.29\cdot 10^{-3}$	$6.11\cdot 10^{-4}$	$7.76\cdot 10^{-5}$	$2.16\cdot 10^{-3}$
$\delta_{T_{r}}$	$2.31\cdot 10^{-1}$	$2.31\cdot 10^{-1}$	$2.31\cdot 10^{-1}$	$2.31\cdot 10^{-1}$
$\bar{A_{T_{N}}}$	$2.63\cdot 10^{1}$	$1.85\cdot 10^{1}$	$3.81\cdot 10^{1}$	$3.46\cdot 10^{1}$
$\delta_{T_{N}}$	$9.49\cdot 10^{-4}$	$9.49\cdot 10^{-4}$	$9.49\cdot 10^{-4}$	$9.49\cdot 10^{-4}$
$\bar{\lambda_{MT_{h}}}$	$6.60\cdot 10^{-3}$	$1.20\cdot 10^{-2}$	$1.33\cdot 10^{-2}$	$1.56\cdot 10^{-2}$
$\bar{\lambda_{MI_{\gamma }}}$	$6.60\cdot 10^{-3}$	$6.57\cdot 10^{-3}$	$1.09\cdot 10^{-3}$	$2.95\cdot 10^{-3}$
$\bar{\lambda_{MIL_{10}}}$	$6.60\cdot 10^{-3}$	$1.24\cdot 10^{-3}$	$5.37\cdot 10^{-3}$	$1.26\cdot 10^{-3}$
$\delta_{M}$	$1.98\cdot 10^{-2}$	$1.98\cdot 10^{-2}$	$1.98\cdot 10^{-2}$	$1.98\cdot 10^{-2}$
$\bar{A_{M_{N}}}$	$3.96\cdot 10^{-2}$	$3.96\cdot 10^{-2}$	$3.96\cdot 10^{-2}$	$3.96\cdot 10^{-2}$
$\delta_{M_{N}}$	$1.98\cdot 10^{-2}$	$1.98\cdot 10^{-2}$	$1.98\cdot 10^{-2}$	$1.98\cdot 10^{-2}$
$\bar{\lambda_{DH}}$	7.06	6.28	6.33	6.35
$\bar{\lambda_{DC}}$	7.06	6.17	6.19	6.03
$\bar{\delta_{DC}}$	$1.39\cdot 10^{1}$	$1.22\cdot 10^{1}$	$1.22\cdot 10^{1}$	$1.21\cdot 10^{1}$
$\delta_{D}$	$2.77\cdot 10^{-1}$	$2.77\cdot 10^{-1}$	$2.77\cdot 10^{-1}$	$2.77\cdot 10^{-1}$
$\bar{A_{D_{N}}}$	$1.44\cdot 10^{1}$	$1.27\cdot 10^{1}$	$1.28\cdot 10^{1}$	$1.27\cdot 10^{1}$
$\delta_{D_{N}}$	$2.77\cdot 10^{-1}$	$2.77\cdot 10^{-1}$	$2.77\cdot 10^{-1}$	$2.77\cdot 10^{-1}$
$\lambda_{C}$	$4.02\cdot 10^{-3}$	$3.80\cdot 10^{-3}$	$1.03\cdot 10^{-2}$	$9.93\cdot 10^{-3}$
$\bar{\lambda_{CIL_{6}}}$	$8.80\cdot 10^{-4}$	$3.97\cdot 10^{-4}$	$3.96\cdot 10^{-3}$	$3.68\cdot 10^{-3}$
$\bar{C_{0}}$	2.50	2.84	2.83	2.86
$\bar{\delta_{CT_{c}}}$	$2.16\cdot 10^{-7}$	$2.00\cdot 10^{-7}$	$6.76\cdot 10^{-7}$	$6.49\cdot 10^{-7}$
$\bar{\alpha_{T_{c}}}$	$8.90\cdot 10^{-5}$	$3.99\cdot 10^{-5}$	$2.50\cdot 10^{-4}$	$5.16\cdot 10^{-5}$
$\bar{\beta_{C}}$	$3.30\cdot 10^{-5}$	$3.75\cdot 10^{-5}$	$3.73\cdot 10^{-5}$	$3.77\cdot 10^{-5}$
$\bar{\delta_{CI_{\gamma }}}$	$2.02\cdot 10^{-7}$	$1.33\cdot 10^{-7}$	$8.87\cdot 10^{-7}$	$1.37\cdot 10^{-6}$
$\delta_{C}$	$2.94\cdot 10^{-3}$	$2.72\cdot 10^{-3}$	$9.22\cdot 10^{-3}$	$8.85\cdot 10^{-3}$
$\bar{\alpha_{NC}}$	3.00	3.00	3.00	3.00
$\delta_{N}$	$8.82\cdot 10^{-3}$	$8.17\cdot 10^{-3}$	$2.77\cdot 10^{-2}$	$2.66\cdot 10^{-2}$
$\bar{\lambda_{I_{\gamma }T_{c}}}$	$1.51\cdot 10^{1}$	$1.95\cdot 10^{1}$	$5.15\cdot 10^{-1}$	8.40
$\bar{\lambda_{I_{\gamma }T_{h}}}$	3.03	5.07	1.77	$2.00\cdot 10^{1}$
$\bar{\lambda_{I_{\gamma }D}}$	$1.51\cdot 10^{1}$	8.73	$3.10\cdot 10^{1}$	4.87
$\delta_{I_{\gamma }}$	$3.33\cdot 10^{1}$	$3.33\cdot 10^{1}$	$3.33\cdot 10^{1}$	$3.33\cdot 10^{1}$
$\bar{\lambda_{HT_{c}}}$	$7.50\cdot 10^{-1}$	1.65	$2.49\cdot 10^{-1}$	$7.44\cdot 10^{-1}$
$\bar{\lambda_{HT_{r}}}$	$7.50\cdot 10^{-1}$	$8.01\cdot 10^{-1}$	$3.25\cdot 10^{-1}$	$4.27\cdot 10^{-1}$
$\bar{\lambda_{HT_{h}}}$	$7.50\cdot 10^{-1}$	2.15	4.27	8.88
$\bar{\lambda_{HN}}$	7.50	6.51	6.17	3.77
$\bar{\lambda_{HM}}$	$7.50\cdot 10^{-1}$	$3.90\cdot 10^{-1}$	$8.25\cdot 10^{-1}$	$4.03\cdot 10^{-1}$
$\bar{\lambda_{HC}}$	7.50	6.51	6.17	3.77
$\delta_{H}$	$1.80\cdot 10^{1}$	$1.80\cdot 10^{1}$	$1.80\cdot 10^{1}$	$1.80\cdot 10^{1}$
$\bar{\lambda_{IL_{10}T_{h}}}$	1.16	2.01	$9.69\cdot 10^{-1}$	3.92
$\bar{\lambda_{IL_{10}T_{c}}}$	1.16	1.55	$5.65\cdot 10^{-2}$	$3.29\cdot 10^{-1}$
$\bar{\lambda_{IL_{10}D}}$	1.16	$6.94\cdot 10^{-1}$	3.41	$1.91\cdot 10^{-1}$
$\bar{\lambda_{IL_{10}M}}$	1.16	$3.66\cdot 10^{-1}$	$1.87\cdot 10^{-1}$	$1.78\cdot 10^{-1}$
$\delta_{IL_{10}}$	4.62	4.62	4.62	4.62
$\bar{\lambda_{I_{2}T_{c}}}$	$4.48\cdot 10^{-1}$	$5.23\cdot 10^{-1}$	$2.26\cdot 10^{-2}$	$8.62\cdot 10^{-2}$
$\bar{\lambda_{I_{2}T_{h}}}$	$8.95\cdot 10^{-1}$	1.36	$7.76\cdot 10^{-1}$	2.06
$\bar{\lambda_{I_{2}D}}$	$4.48\cdot 10^{-1}$	$2.34\cdot 10^{-1}$	1.36	$5.00\cdot 10^{-2}$
$\bar{\lambda_{I_{2}M}}$	$4.48\cdot 10^{-1}$	$1.23\cdot 10^{-1}$	$7.50\cdot 10^{-2}$	$4.66\cdot 10^{-2}$
$\delta_{I_{2}}$	2.24	2.24	2.24	2.24
$\bar{\lambda_{IL_{6}C}}$	$3.57\cdot 10^{-1}$	$2.46\cdot 10^{-1}$	$4.71\cdot 10^{-2}$	$5.89\cdot 10^{-2}$
$\bar{\lambda_{IL_{6}M}}$	$2.38\cdot 10^{-1}$	$9.82\cdot 10^{-2}$	$4.20\cdot 10^{-2}$	$4.20\cdot 10^{-2}$
$\bar{\lambda_{IL_{6}T_{h}}}$	$2.38\cdot 10^{-1}$	$5.40\cdot 10^{-1}$	$2.17\cdot 10^{-1}$	$9.24\cdot 10^{-1}$
$\bar{\lambda_{IL_{6}D}}$	$2.38\cdot 10^{-1}$	$1.86\cdot 10^{-1}$	$7.64\cdot 10^{-1}$	$4.50\cdot 10^{-2}$
$\delta_{IL_{6}}$	1.07	1.07	1.07	1.07
